# Interactions of the male contraceptive target EPPIN with semenogelin-1 and small organic ligands

**DOI:** 10.1038/s41598-023-41365-1

**Published:** 2023-09-01

**Authors:** Antoniel A. S. Gomes, Natália C. M. Santos, Leonardo R. Rosa, Rafael J. Borges, Marcos R. M. Fontes, Katherine G. Hamil, Michael G. O’Rand, Erick J. R. Silva

**Affiliations:** 1 Department of Biophysics and Pharmacology, Institute of Biosciences of Botucatu, São Paulo State University, Botucatu, SP Brazil; 2 The Center of Medicinal Chemistry (CQMED), Center for Molecular Biology and Genetic Engineering (CBMEG), University of Campinas (UNICAMP), Campinas, Brazil; 3 Institute for Advanced Studies of the Sea (IEAMAR), São Paulo State University, UNESP, São Vicente, SP Brazil; 4 Research and Development, Eppin Pharma Inc., Chapel Hill, NC USA; 5 Department of Cell Biology and Physiology, University of North Carolina at Chapel Hill, Chapel Hill, NC USA; 6 Present Address: Laboratory of Biological Physics, Carlos Chagas Filho Institute of Biophysics, Universidade Federal do Rio de Janeiro, Rio de Janeiro, RJ Brazil

**Keywords:** Protein analysis, Pharmacology, Molecular modelling, Reproductive biology

## Abstract

Novel male contraceptives will promote gender equality in sharing contraceptive responsibility. The sperm-associated protein epididymal protease inhibitor (EPPIN) is a promising target for non-hormonal male contraception. EPPIN interacts with the semen coagulum protein semenogelin-1 (SEMG1) on the sperm surface, leading to transient inhibition of sperm motility after ejaculation. Small organic molecules targeting EPPIN's SEMG1-binding are under development as male contraceptives. Here, we combined computational approaches to uncover key aspects underlying EPPIN binding to SEMG1 and small organic ligands. We generated a human EPPIN model showing a typical arrangement of the WFDC (Whey-acid four disulfide core)-type and Kunitz-type domains, connected by a hinge region. Determining the EPPIN model's intrinsic motion by molecular dynamics simulations and normal mode analysis revealed a conformation, presenting a binding pocket that accommodates SEMG1^Glu229-Gln247^, EP055, and EP012. EPPIN's residues Phe63 and Lys68 (WFDC domain), Asp71 (hinge region), and Asn113, Asn114, and Asn115 (Kunitz domain) were identified as hot spots for SEMG1, EP055, and EP012 binding. Moreover, hydrophobic and hydrophilic residues in the WFDC and Kunitz domains allow plasma membrane anchoring, orienting the EPPIN binding pocket to the solvent. Targeting EPPIN's essential residues for its biomolecular interactions may improve the rational design of EPPIN ligands as spermiostatic compounds.

## Introduction

Contraceptive methods are an array of approaches that reversibly inhibit fertility, thereby decreasing the chances of pregnancy after sexual intercourse^1^. Currently, there are several contraceptive methods for women, who are the main carrier of contraceptive responsibility and family planning^2^. Despite this trend, almost half of pregnancies worldwide are unintended, putting women at risk of pregnancy-related health issues and imposing negative social and economic impacts on their families^3^. Moreover, over 100 million women worldwide desire to prevent pregnancies but do not use any contraceptive option^4^. The limited options of male contraceptive methods (condoms and vasectomy) further contribute to the unintended pregnancy rate and reduced engagement of male partners in family planning. Given studies demonstrating the willingness of men to increase their participation in family planning, the introduction of novel male contraceptive methods in the market can significantly improve the current landscape, thus contributing to the improvement of family planning and reducing unintended pregnancies^5–7^.

Pharmacological strategies for non-hormonal male contraception may target the testis (sperm production), the epididymis/vas deferens (sperm maturation or delivery), or the mature spermatozoon itself^8^. The latter is based on loss-of-function and provides drug targets that promote inhibition of male fertility with fast onset and reversibility. Among the sperm-based targets for male contraceptive drug development, the sperm protein EPPIN (epididymal protease inhibitor) is a promising candidate^9^.

EPPIN belongs to the WFDC (whey-acidic protein four-disulfide core) protease inhibitor family, displaying an N-terminal WFDC domain and a C-terminal Kunitz-type protease inhibitor domain^10^. EPPIN is a sperm surface node for protein–protein interactions with crucial roles in providing antimicrobial protection for spermatozoa and modulating their function as they travel in the female reproductive tract^11^. EPPIN interacts with seminal plasma proteins such as protease prostate-specific antigen (PSA) and semenogelin-1 (SEMG1) on the surface of ejaculated spermatozoa^12–14^. SEMG1 is the most abundant component of the semen and an endogenous inhibitory factor of sperm motility^15^. EPPIN/SEMG1 binding on the sperm surface inhibits sperm motility^16,17^.

EPPIN's SEMG1-binding site has been successfully used as a docking site to develop molecules that bind spermatozoa and display spermiostatic activity in both human and animal models^11,18^. Anti-EPPIN antibodies targeting the epitope S21C (S21C-Ab, S^103^MFVYGGCQGNNNNFQSKANC^123^; part of the Kunitz domain) exhibit in vitro spermiostatic activity in human and mouse spermatozoa and induce reversible infertility in monkeys^19–21^. S21C-Ab impaired sperm function via internal pH acidification and Ca^2+^ loss^19^. High throughput screening applications resulted in the identification of small organic compounds, such as EP012, and EP055, that substitute for SEMG1 and the anti-EPPIN antibodies leading to the inhibition of motility in human spermatozoa and non-human primate models^11,18,22^.

A better understanding of the structural aspects of EPPIN/SEMG1 interaction will facilitate the rational optimization of lead compounds and expand the knowledge of the underlying mechanisms by which protein–protein interactions govern sperm function and male fertility. No crystal structures of EPPIN and SEMG1 have been reported in public databases such as PDB (https://www.rcsb.org/)^23^. Using experimental and computational approaches, we and others have previously shown that EPPIN's residues within the Tyr107-Phe117 loop in the S21C epitope, such as Tyr107 and the Asn repeat (Asn113 to Asn116), interact with SEMG1's residues and are likely to interact with EPPIN ligands^11,24,25^. These studies further revealed that residues upstream and downstream of the Tyr107-Phe117 loop, such as Arg32, Gln118, and Lys120, could play a role in EPPIN binding SEMG1^11,24^. To provide further insights into the EPPIN structural aspects, we combined molecular dynamics (MD) simulations and normal mode analysis (NMA) to perform a conformational exploration of the full-length EPPIN homology model, determining its intrinsic motions and stable conformations. We further submitted these conformations to molecular docking followed by MD simulations to determine the contribution of EPPIN residues in the interaction with endogenous (SEMG1) and exogenous (EP055 and EP012) ligands. Our modeling analyses identified that residues in the EPPIN WFDC and Kunitz domains form a binding pocket that can accommodate such ligands. We further determined that a hinge segment (Asp71 to Asp75) connecting these domains is essential for stabilizing ligands in the binding pocket. Moreover, we observed that hydrophobic and hydrophilic residues in both WFDC and Kunitz domains are important for EPPIN's anchoring in the plasma membrane, thereby maintaining the binding pocket turned to the solvent for the docking of SEMG1 or small ligands.

## Results

### Modeling and conformational exploration of the full-length EPPIN model

**Figure 1:**
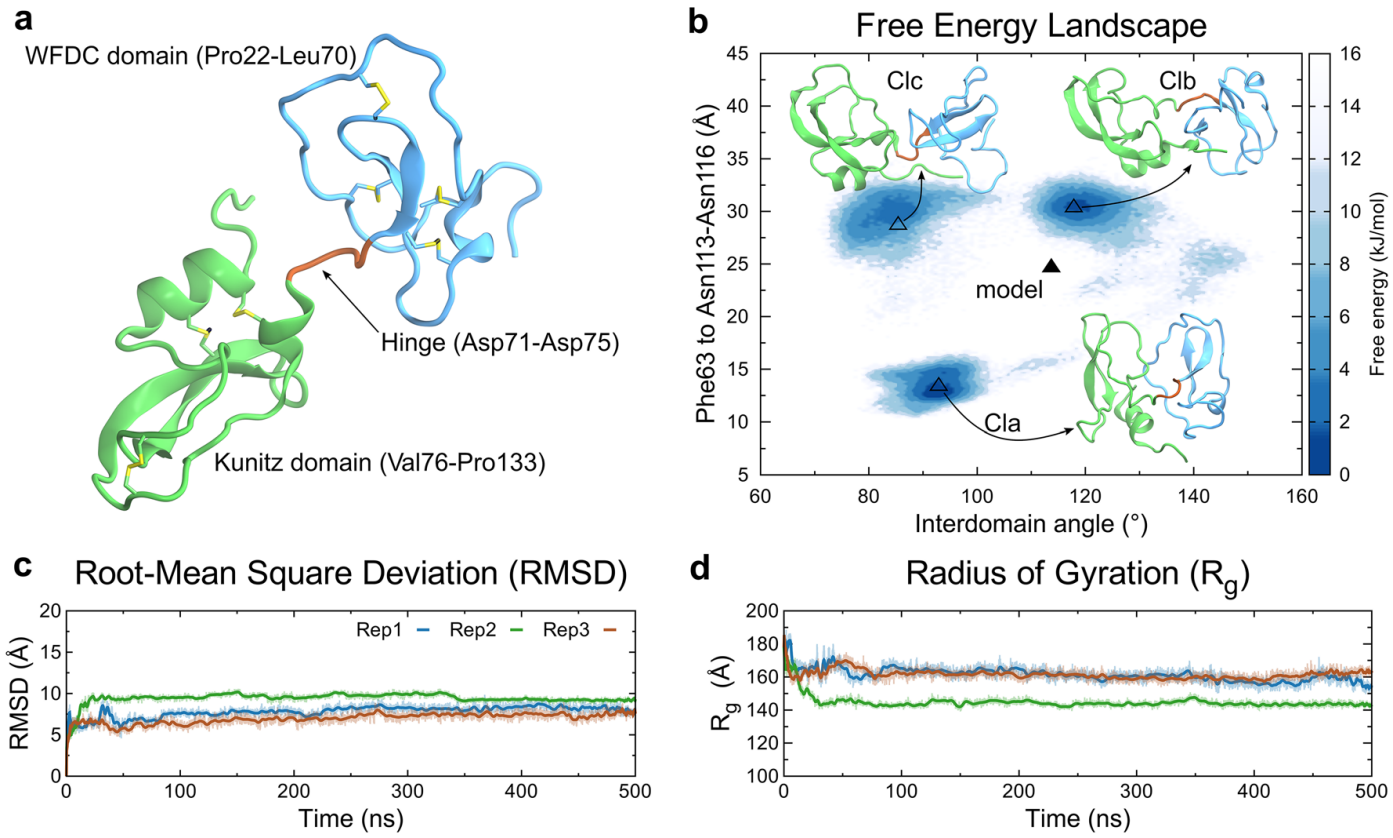
Structural aspects of the full-length EPPIN model. (**a**) The initial model of EPPIN generated by homology modeling, highlighting the N-terminal, the hinge segment, and the C-terminal domains shown as a cartoon in blue, brown, and green, respectively. Disulfide bonds are presented as sticks, with sulfur in yellow. (**b**) Gibbs free energy landscape of the model after conformational exploration of the three independent replicas of MD simulations, using the distances of Phe63 to the Asn repeat and the interdomain angle (θ) as variables to classify all conformations. Three states were observed, with representative conformations named Cla, Clb, and Clc, shown by empty triangles. The initial structure (model) is shown as a filled triangle. (**c**) Temporal RMSD and (**d**) Rg calculations for each replica are shown in blue, green, and brown.

We generated a full-length EPPIN (mature sequence, Pro22 to Pro133; UniProtKB—O95925) model by homology modeling mixing different experimental structures that covered the WFDC or Kunitz domains (Supplementary Table S1). The generated model was geometrically validated using the Ramachandran analysis available in the MolProbity server, which identified 99.1% of all residues in allowed regions (Supplementary Fig. S1). The model presented a classical WFDC-type and a Kunitz-type folding for the N-terminal domain (Pro22 to Leu70) and the C-terminal domain (Val76 to Pro133) (Fig. 1a). These domains were connected by a hinge region formed by the segment of residues from Asp71 to Asp75 (Fig. 1a). The model presented seven disulfide bonds, with four bonds located in the WFDC domain (Cys33-Cys61, Cys40-Cys65, Cys48-Cys60, and Cys54-Cys69) and three bonds in the Kunitz domain (Cys77-Cys127, Cys86-Cys110, and Cys102-Cys123) (Fig. 1a).

We submitted the EPPIN model to MD simulations to explore its conformational space to obtain stable and more energetically favorable structures. Frames of all three replicas of 500 ns were analyzed together to generate a free-energy landscape plot. The distance of Phe63 to the Asn repeat (center of mass of C⍺ residues range Asn113–Asn116) and an interdomain angle (θ), measured by the center of mass of C⍺ residues (i) Cys60-Val62 and Lys67-Cys69; (ii) Lys120-Asn129; and (iii) Phe90-Asp96 and Asn100-Tyr107 were used as reaction coordinates to classify the set of structures (Fig. 1b and Supplementary Fig. S2). We clusterized all conformations obtained from all three replicas to obtain representative central structures of each state, hereby named Cla, Clb, and Clc (Fig. 1b). These three clusters corresponded to around 85% of all structures, with Cla, Clb, and Clc presenting 33.2, 29.6, and 22.2%, respectively. The main state, Cla, presented a packed set of structures, with the distance of Phe63 to the Asn repeat of 13.4 Å, while the other two states, Clb and Clc, presented values of 30.4 and 28.7 Å, respectively. The interdomain angle was reduced in Cla and Clc, with values of 92.9° and 85.4°, respectively, and Clb presented a value of 117.9°.

The time-evolution RMSD showed that each replica converges to a specific conformation (Fig. 1c), in which all tended to assume a closed conformation between the WFDC and Kunitz domain interaction, connected by an interdomain hinge segment (Fig. 1b, brown segment). In addition, these conformations presented a globular form in comparison to the model, evidenced by the radius of gyration (Rg) below 178 Å (model), with Cla presenting the lowest value, 144 Å (Fig. 1d). The evolution of RMSD and Rg values for each replica is shown in Fig. 1c, d, indicating that each replica converged to a particular state.

NMA confirmed the ability of the EPPIN model to assume a closed conformation, with such a motion being described by the lowest-frequency normal (mode 7) (Supplementary Fig. S3). This result reinforces that the WFDC and Kunitz domains interact to access a compact conformation. Normal modes 8 and 9 were also analyzed, as they presented lateral movements that could play a role in the accommodation of the WFDC and Kunitz domains (Supplementary Fig. S3). To test if MD simulations followed the lowest-frequency normal mode directions, we obtained the main MD eigenvector (called mode 1), the most relevant global movement explored in MD, of each replica and correlated them to normal mode vectors 7 to 9. Mode 7 was explored by replica 2, corresponding to the closed conformation observed in Cla, with a moderate correlation (R = 0.58) (Supplementary Fig. S3). Modes 8 and 9 were poorly explored during MD simulations, with replica 1 presenting a weak correlation to mode 8 (R = − 0.29) and replica 3 presenting a weak negative correlation (R = − 0.36) with mode 7 (Supplementary Fig. S3). Despite the potential relevance of Cla, we selected all three central clusters, as they may be of structural importance to EPPIN's physiological function as a modulator of sperm motility to identify druggable hot spots.

### EPPIN model druggable hot spots

**Figure 2:**
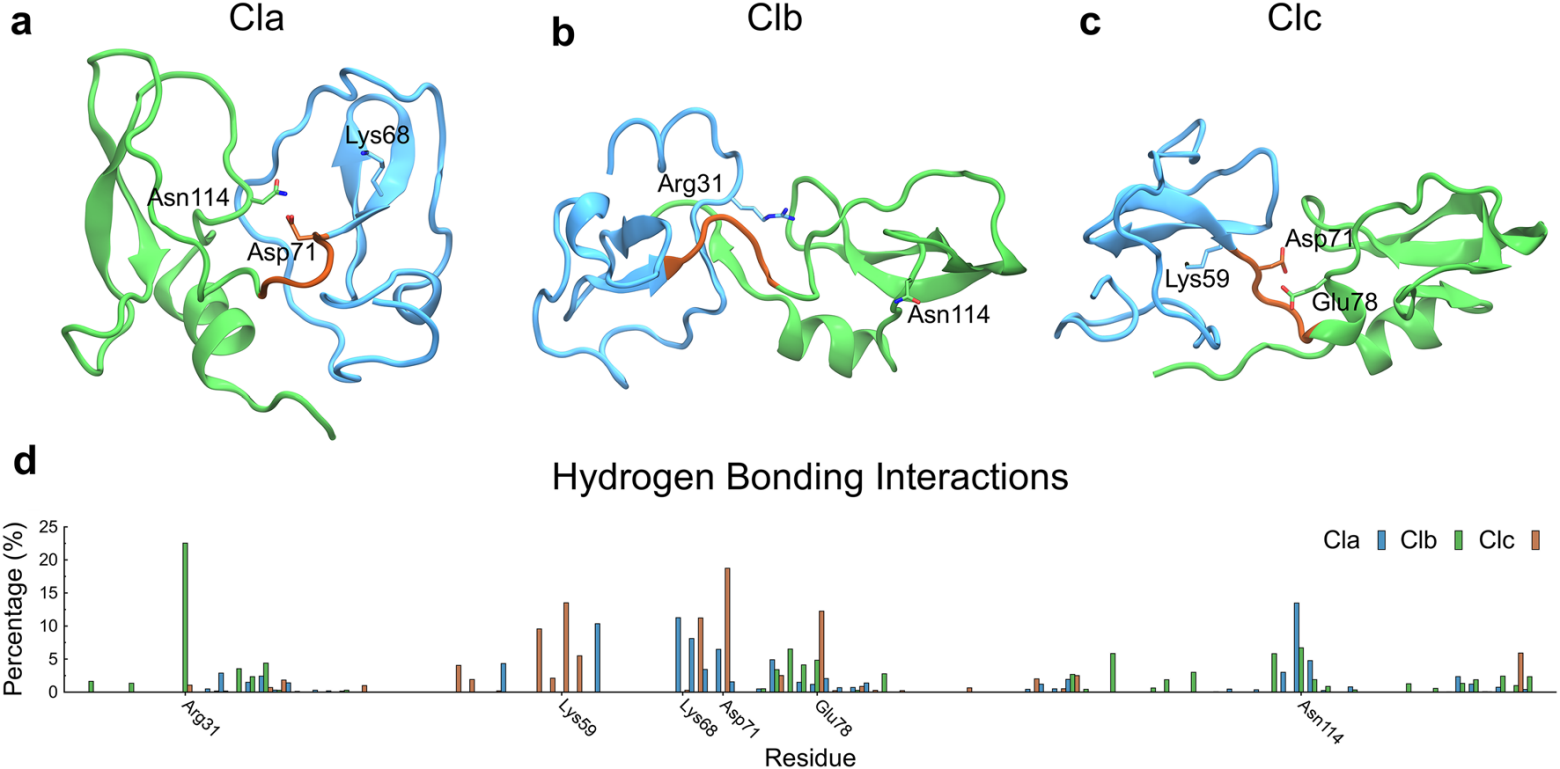
FTMap of the three main states of the EPPIN model. Cla, Clb, and Clc were used for calculations to determine the most important residues for hydrogen bonding interactions. The upper panel shows the states (**a**) Cla, (**b**) Clb, and (**c**) Clc, presenting the position of residues with a high percentage to form hydrogen bonds to ligands. (**d**) The lower panel shows the percentage of hydrogen bonding interactions with ligands for each residue of Cla (blue bars), Clb (green bars), and Clc (brown bars). WFDC (blue) and Kunitz (green) domains and hinge segment (brown) are shown.

Once the conformational space of the EPPIN model was explored by MD simulations, we submitted its three central cluster conformations to the FTMap server to identify possible druggable regions (Supplementary Fig. S4). Then, we analyzed such structures to identify the main residues able to form hydrogen bonds (Fig. 2). Regarding the Cla conformation, Lys68, Asp71, and Asn114 were identified as important residues, given their ability to form hydrogen bonds to the set of molecules used by the server, corresponding to 11.3, 6.5, and 13.5%, respectively (Fig. 2a, d). Following, Clb showed Arg31 (22.5%) and Asn114 (6.7%) as important residues for hydrogen bonding (Fig. 2b, d), and Clc presented Lys59, Asp71, and Glu78, with percentages for the same parameter of 13.5, 18.7, and 12.3%, respectively (Fig. 2c, d). We observed druggable regions in both WFDC and Kunitz domains and the hinge region near the Asp71 residue (Fig. 2d). In addition, the residue Asn114, which is part of the Asn repeat in the Kunitz domain, was identified as an important residue in Cla and Clb, emphasizing the importance of this region in EPPIN activity. These results suggest that EPPIN's Kunitz residue Asn114 and the interdomain residue Asp71 are likely part of a binding pocket for molecules.

### Interaction between EPPIN and SEMG1

We submitted all three central cluster conformations to molecular docking against SEMG1^Glu229-Gln247^ (SEMG1-E2Q peptide) to identify their binding interface using the Patchdock server^26^. The SEMG1-E2Q peptide has been previously shown to dock the EPPIN Kunitz domain and identified as the minimal SEMG1 sequence required to inhibit sperm motility^11,17^. For each cluster, the two best dockings were selected and submitted to MD simulation to analyze their stability during 100 ns using the RMSD evolution of backbone atoms of SEMG1 as a parameter, aligning the complex on EPPIN's backbone atoms (Supplementary Fig. S5). The initial (docking) and final (after 100 ns of MD simulations) conformation for each MD simulation showed that the SEMG1-E2Q peptide moved away from the Asn repeat or lost its conformation, in particular its helices, for all cases, except for Docking 1 of Cla and Clc (Supplementary Fig. S5); therefore, we extended the MD simulations to 500 ns of these two complexes.

**Figure 3:**
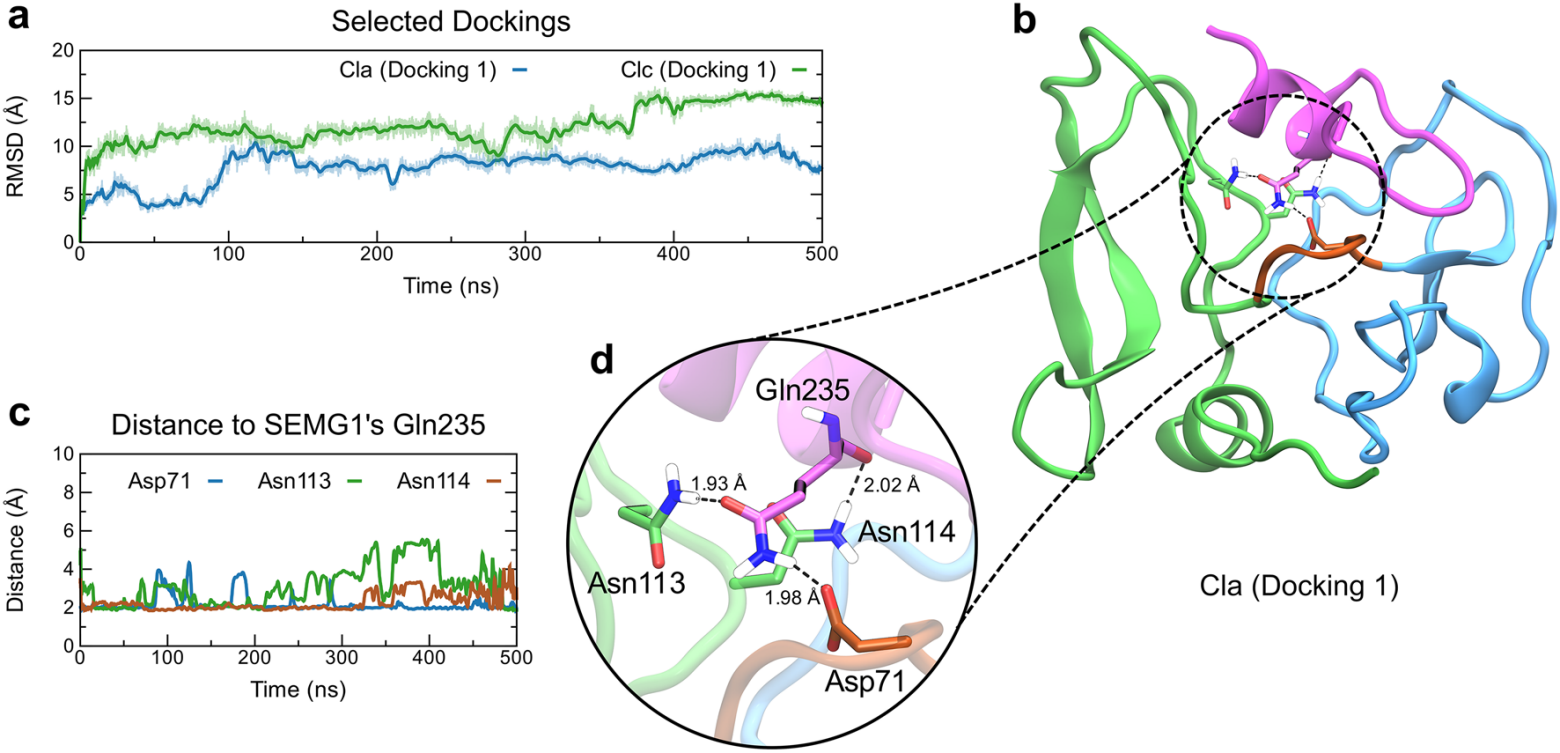
Interaction between the full-length EPPIN model with the endogenous ligand SEMG1. (**a**) Docking 1 of Cla (blue line) and Clc (green line) during 500 ns of MD simulation. (**b**) The final orientation of Docking 1 of Cla with EPPIN is shown in detail. EPPIN WFDC domain (blue), Kunitz domain (green) and hinge segment (brown), and SEMG1-E2Q peptide (purple) are shown. (**c**) Time evolution of the minimal distance between EPPIN Asp71 (blue line), Asn113 (green line), and Asn114 (brown line), and SEMG1 Gln235 is shown. (**d**) The spatial orientation of such residues is shown in detail.

The analysis of RMSD values of these MD simulations showed that Docking 1 of Cla presented lower values in comparison to Docking 1 of Clc (Fig. 3a), indicating that the former converged to a stable state. To verify this possibility, we further performed RMSD calculations of backbone atoms of the complex using the average structure as a reference for each case. We observed that Docking 1 of Cla converged near the average structure from 150 ns in a region of RMSD around 3.5 Å (Supplementary Fig. S6). On the other hand, Docking 1 of Clc started moving away from the average structure after 200 ns (Supplementary Fig. S6), suggesting that this complex would require more time than 500 ns to be stabilized or assume another conformation. In addition, Docking 1 of Clc showed SEMG1-E2Q peptide variable loop in some regions, being in contact with the WFDC domain, reinforcing that the complex EPPIN/SEMG1 should present a different orientation (Supplementary Fig. S7).

These results pointed to Docking 1 of Cla as a potential candidate complex to describe the interaction of EPPIN and SEMG1 (Fig. 3a); therefore, we analyzed this complex in detail. After 500 ns of MD simulations, the structure of the complex maintained the SEMG1-E2Q peptide's helical folding, placed in the binding pocket formed by the WFDC and Kunitz domains (Fig. 3b). To analyze the interactions between EPPIN and SEMG1-E2Q peptide, we calculated the percentage of intermolecular contacts in the complex during the MD simulations (Supplementary Table S2). Consistently with the critical roles of EPPIN's Kunitz domain to the interaction of SEMG1, residues in the Asn repeat in the Kunitz domain Asn113, Asn114, and Asn115 interacted with the SEMG1-E2Q peptide during 83.8%, 98.1%, and 80.7% of the simulated time, respectively (Fig. 3c, d; Supplementary Table S2). Interestingly, EPPIN residue Asp71, in the hinge region, also interacted with the peptide during 94.5% of the simulated time (Fig. 3c, d; Supplementary Table S2).

To obtain more detailed information on the main SEMG1-E2Q peptide residues for stabilizing the complex, we generated a matrix of the percentage of contacts (Supplementary Table S3) and a diagram of types of interaction (Supplementary Fig. S10). We observed that the SEMG1's Gln235 residue plays an important role in EPPIN interaction via hydrogen or Van Der Waals bonds, as shown by its strong interaction with Asp71 (93.2%) and the Asn repeat residues in the Kunitz domain Asn113 (66.2%), Asn114 (94.5%) and Asn115 (88.5%). Moreover, SEMG1's Cys239 main chain oxygen showed important contacts with the WFDC domain by forming a hydrogen bond with EPPIN's Lys68 side chain nitrogen during 87% of the simulated time (Supplementary Fig. S10 and Supplementary Table S3). We also identified the presence of water molecules in the interface between EPPIN and SEMG1, which were located around EPPIN’s Asn repeat and Lys68 residue, likely contributing to stabilizing the complex (Supplementary Fig. S8).

### Interaction between EPPIN and small organic ligands (EP055 and EP012)

We submitted the EPPIN model Cla, Clb, and Clc structural clusters to a docking analysis to lead compounds experimentally identified as EPPIN ligands and inhibitors of sperm motility (EP055 and EP012)^18^. For that, we used the DockThor server^27^ to predict the interaction of EPPIN with EP055 and EP012.

**Figure 4:**
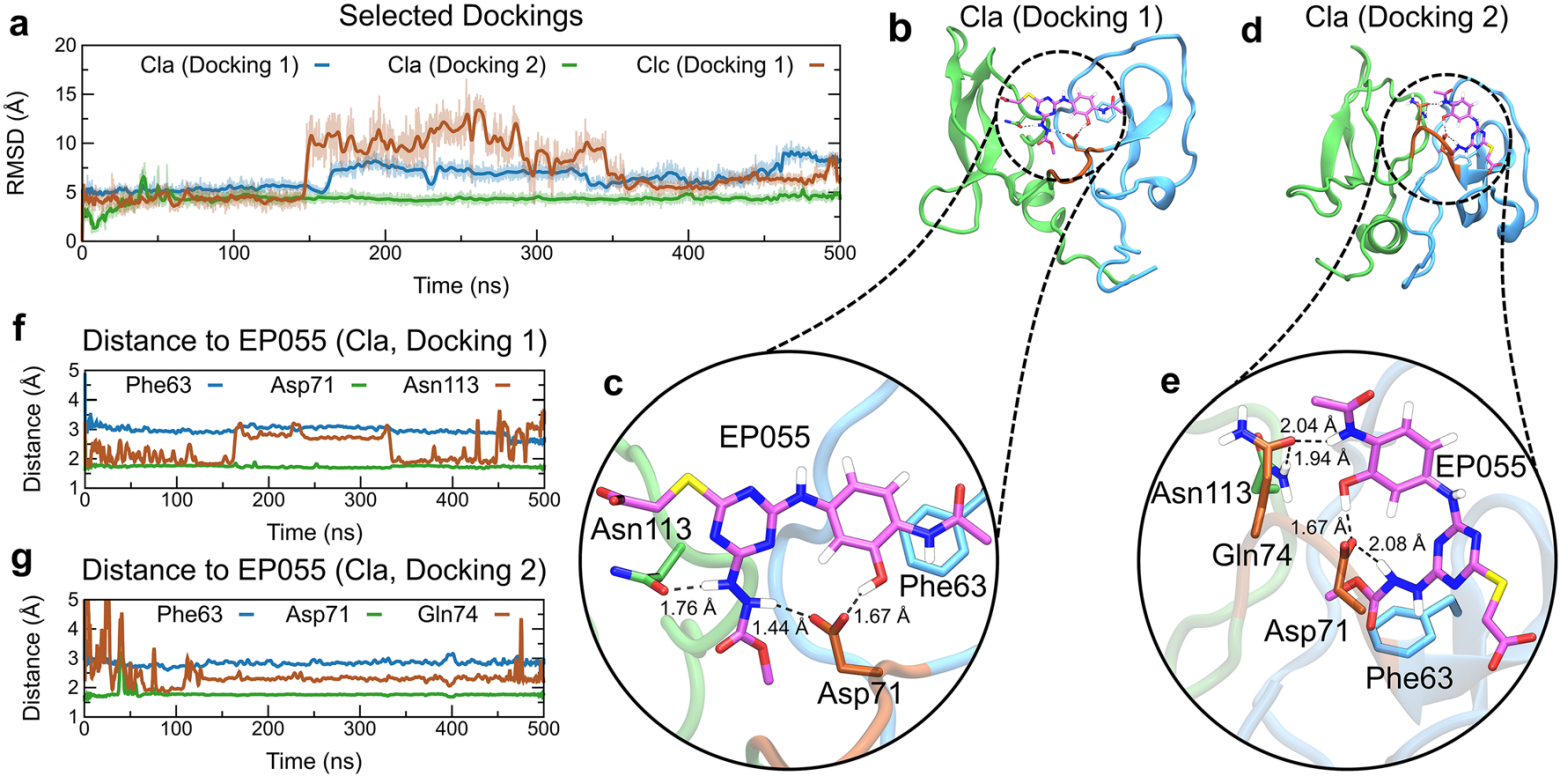
Interaction between the full-length EPPIN model with the exogenous ligand EP055. (**a**) Temporal RMSD of Docking 1 of Cla (blue line), Docking 2 of Cla (green line), and Docking 1 of Clc (brown line) during 500 ns of MD simulation. (**b**) Docking 1 and (**d**) Docking 2 of Cla with EPPIN are shown. EPPIN WFDC domain (blue), Kunitz domain (green) and hinge segment (brown), and EP055 ligand (purple) are shown. Time evolution of the minimal distance between important residues of EPPIN for (**f**) Docking 1 and (**g**) are shown, with (**c,e**) their spatial orientation shown in detail.

To select the best poses, we considered the spatial orientation near the binding pocket identified by FTMap (Fig. 2) and the PatchDock binding energies of the complexes. The two best poses for each EPPIN structural cluster were submitted to 100 ns of MD simulations to check the conformational stability of the complex. Similar to what we observed for SEMG1 docking, Cla showed spatial stability of EP055 during 100 ns, with RMSD values below 5 Å for both dockings (Supplementary Fig. S9). In addition, Docking 1 of Clc also showed similar behavior (Supplementary Fig. S9). Therefore, both dockings of Cla and Docking 1 of Clc were extended until 500 ns of MD simulation for further structural inspection (Fig. 4a).

After 500 ns of MD simulations, Docking 1 of Clc showed that EP055 moved away from the Kunitz domain, located deep in the WFDC domain, and interacted mainly with the hinge region (Supplementary Fig. S7). Moreover, the compound did not present interactions with the Asn repeat in the EPPIN Kunitz domain, indicating that this conformation cannot corroborate the experimental results^18^. Conversely, both dockings of Cla showed EP055 interacting with both domains of EPPIN, and despite a different ligand orientation, they interact with similar residues (Fig. 4b, d). In both cases, we observed that the hinge region, particularly the Asp71 residue, strongly interacts with EP055, forming hydrogen bonds between the hydroxyl and the diazene groups of the ligand (Fig. 4c). Docking 1 of Cla showed strong interactions with Kunitz domain residues, such as Asn113 and Asn114 (Fig. 4b, c, f). On the other hand, Docking 2 of Cla interacted mainly with the WFDC domain, with the hinge-residues Asp71 and Gln74 mediating hydrogen bonds with polar atoms of EP055 (Fig. 4d–e, g). We further observed a hydrophobic contact between EP055 and Phe63 in both dockings (Fig. 4c, e, g), suggesting that the N-domain can interact with hydrophobic groups, such as benzene, methyl, and methyl ester groups; all of them found in the EP055 structure.

The percentage contacts between EP055 and important residues of EPPIN for each docking of Cla and Clc during MD simulation is presented in Supplementary Table S4. EPPIN Phe63 and Asp71 residues were the main residues in contact with EP055 in Cla, presenting values of 94.2% and 99.7% for Docking 1 and 97.3% and 99.9% for Docking 2, respectively (Supplementary Table S4). Regarding the Asn repeat in the EPPIN Kunitz domain, only Docking 1 showed a high percentage interaction with Asn113 and Asn114 (97.3% and 98.6%, respectively), whereas Docking 2 of Cla showed lower values (40.3% and 30.7%, respectively) (Supplementary Table S4). Further, Phe63 and the Asn repeat present types of close interaction in common between Docking 1 and Docking 2 of Cla (Supplementary Fig. S10). We also identified one water molecule in the interface of the complex EPPIN/EP055 in Docking 2, interacting with EPPIN’s Asp71 and Asn113 residues and EP055’s methyl ester group (Supplementary Fig. S8). Interestingly, such water was not observed in Docking 1, which was occupied by the EP055’s diazene group (Fig. 4c).

As both orientations presented interactions around the Asn repeat and the hinge region, we calculated the binding energy of each complex using the molecular mechanic energies combined with the Poisson–Boltzmann surface area method (MM/PBSA)^28^. Frames from the last nanosecond were used in the calculations, resulting in similar binding energies for Docking 1 and 2, with values of −33.7 ± 6.1 and −39.3 ± 5.2 and kcal/mol, respectively. Thus, according to experimental results showing that EP055 presents a stronger interaction with the Kunitz domain of EPPIN^18^, we consider that both orientations could be assessed in the EPPIN structure.

EP012, the other EPPIN ligand analyzed, presents a similar structure to EP055, with the presence of a secondary methyl ester instead of a methyl group in the benzyl group^18^. As observed in Docking 1 and 2 of Cla, EPPIN accommodated these same substitutions in both WFDC and Kunitz domains (Fig. 4c, e). Thus, we decided to analyze if EP012 could be accommodated in EPPIN and check the structural similarities of the complex compared to EP055. For this end, we selected a conformation of the EPPIN/EP055 complex, as observed in Fig. 4c (see also Supplementary Fig. S11), to dock EP012 on EPPIN. As expected, EP012 presented a conformation similar to the one observed for EP055 (Supplementary Fig. S11). Consistently, the EP055/EPPIN and EP012/EPPIN complexes presented similar predicted free energy values by DockThor (−7.43 kcal/mol and −7.45 kcal/mol, respectively)^27^. The binding region observed for both ligands overlaps in the SEMG1 peptide binding (Supplementary Fig. S11).

### MD simulations suggest that EPPIN is anchored in the plasma membrane through both hydrophobic and hydrophilic interactions

After selecting the most stable conformation of EPPIN by MD simulations (Fig. 1) and further molecular docking experiments against SEMG1-E2Q peptide (Fig. 3) and EP055 (Fig. 4), we selected the conformation of the complex EPPIN/SEMG1 to analyze its ability to interact with membranes using the OPM server^29^. We found that EPPIN can accommodate on the membrane surface by hydrophobic residues in the WFDC domain (Leu24) and the Kunitz domain (Phe123 and Pro133), with an energy value of −6.3 kcal/mol and depth of 3.4 ± 0.6 Å.

**Figure 5:**
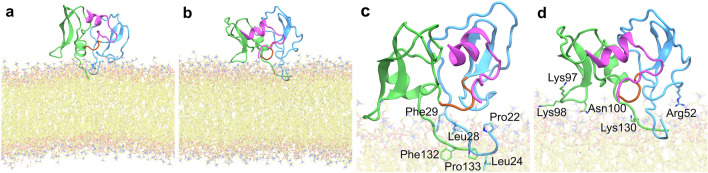
Interaction model of the full-length EPPIN model with the plasma membrane. The complex EPPIN/SEMG1 peptide was selected for anchoring in a 100% POPC bilayer. EPPIN WFDC domain (blue), Kunitz domain (green) and hinge segment (brown), SEMG1-E2Q peptide (purple), and POPC (yellow) are shown. (**a**) The initial orientation of the complex was predicted by the OPM server^28^, with (**b**) its final orientation obtained after 100 ns of MD simulation. (**c**) Hydrophobic and (**d**) polar residues of EPPIN were identified as important in the interaction and anchoring on the plasma membrane.

Based on the OPM orientation, we submitted the EPPIN model to MD simulations using a lipid bilayer composed of phosphatidylcholine (POPC) (Fig. 5). The initial conformation after membrane accommodation around the protein is shown in Fig. 5a. After 100 ns of MD simulation, the EPPIN model maintained the same global orientation as suggested by OPM, with the terminal residues from the WFDC and Kunitz domains interacting with the hydrophobic environment of the membrane (Fig. 5b). Leu24 is a terminal WFDC domain residue surrounded by other residues able to form hydrophobic interactions, such as Pro22, Thr25, Trp27, Leu28, and Leu29; all of them were in contact with the membrane for more than 50% of the time (Fig. 5c; Supplementary Table S5). Regarding the residues from the Kunitz domain, Phe132 and Pro133 were in contact with the membrane during the time length of the simulation, indicating that this region is also likely to be in contact with the membrane (Fig. 5c; Supplementary Table S5). Moreover, we detected important polar contacts between EPPIN residues and phospholipids' heads of POPC. For example, Arg52, Lys97, Lys98, Asn100, and Lys130 residues were in contact with the membrane during > 90% of the simulated time, presenting polar contacts with phospholipid’s heads (Fig. 5d; Supplementary Table S5). These residues are placed in the extremity of both WFDC and Kunitz domains, and are turned to the solvent, facilitating the interaction with the membrane surface and consequent stabilization of EPPIN.

## Discussion

No new contraceptive method for men has been marketed in over a century. In this regard, novel male contraceptives are urgently needed to address the gender inequity in contraceptive responsibility. Unintended pregnancies have a significant negative socio-economic impact on women and their families. Drug development targeting EPPIN could result in the first generation of on-demand male contraceptives targeting sperm function, thereby representing a breakthrough for birth control since the first hormonal pill to women. Understanding EPPIN's structural properties will bring to light states with structural and functional relevance to facilitate its development as a drug target^11^. In the present study, we combined bioinformatics approaches to explore the structure of EPPIN, suggesting relevant conformations, protein–protein and protein–ligand interacting surfaces, and druggable hotspots. We showed that EPPIN converged to a closed conformation, assessed by hinge-bending motion, allowing WFDC and Kunitz domain interactions. This arrangement resulted in an orthosteric binding pocket that interacts with SEGM1 and mimetic compounds through hydrophilic and hydrophobic interactions. These structural aspects support the positioning of EPPIN on the cell membrane and the exposure of its binding pocket. SEMG1, EP055, or EP012 binding in the hinge region could lead to EPPIN's conformation changes, resulting in the modulation of other membrane-associated components (e.g., ion channels) governing sperm function. This hypothesis finds support from observations that EPPIN forms a multiprotein complex via different protein–protein interactions on the sperm surface^12^. Our findings pave the way for the rational design of high-affinity EPPIN ligands with potent spermiostatic activity and higher prospects for effectiveness and safety in preclinical and clinical trials.

MD simulation is an efficient technique to explore the conformational space of proteins to determine stable conformations^30,31^. Since EPPIN has no structure experimentally elucidated, in the present work, we built a model by homology modeling and further analyzed it by MD simulations. We identified three main conformations of EPPIN using MD simulations, with the most populated one presenting a closed conformation assessed by a hinge-bending motion between WFDC and Kunitz domains, which was further reinforced by NMA. Such a motion is commonly described by NMA of other proteins, being important to determine transitional states toward stable conformations^32^ and the interface between domains^33^. The agreement between MD simulations and NMA suggests that EPPIN assumes a stable closed conformation. The progress towards such conformation can be observed in the Supplementary Video S1.

Recently, the structures of several human proteins were predicted by AlphaFold^34^, including EPPIN (code O95925). The AlphaFold EPPIN model was predicted with high confidence, showing that the WFDC and Kunitz domains interact to assume a compact conformation. Such a conformation has a similar fold as observed in Cla, with an interdomain angle 20° lower than Cla (Supplementary Fig. S12). However, when submitted to an MD simulation step of 500 ns, the AlphaFold EPPIN model showed conformational instabilities, resulting in domain dissociation and, consequently, unfolding of its probable binding pocket (Supplementary Fig. S12), which was not observed in MD simulations of the Cla conformation bound to SEMG1 or EP055. Therefore, further studies are necessary to achieve a stable conformation of the EPPIN model predicted by AlphaFold and then verify its interaction with SEMG1 and small molecules.

The closed conformation of EPPIN obtained by MD simulations in the present work showed other interesting features that could be related to its physiological relevance, such as the presence of a druggable hotspot containing the Asn repeat in the Kunitz domain, considered essential for EPPIN activity. Experimental data demonstrated that the loop Tyr107-Phe117, which contains the Asn repeat, is crucial to EPPIN/SEMG1 binding^25^. Thus, we suggest that this pocket is a probable binding site for different molecules, as it accommodated SEMG1-E2Q, EP055, and EP012 ligands. We identified Phe63, Lys68, Asp71, Asn113, Asn114, and Asn115 residues in common for the binding of these molecules, suggesting that EPPIN has a common binding pocket formed by residues from the WFDC (Pro22-Leu70) and Kunitz (Val76-Pro133) domains, and the hinge (Asp71–Asp75) region. In addition, the presence of water molecules in protein binding sites is considered crucial for protein–ligand recognition^35^; thus, the identification of a central water molecule stabilizing the hinge and Asn repeat reinforces the importance of the EPPIN’s binding pocket in ligand recognition and stabilization.

Our current study showed important divergences from a previous work that also performed EPPIN modeling^24^. First, except for Arg32, no residues from the WFDC domain were found in contact with SEMG1 or EP055. Another difference is that our results revealed the crucial role of the hinge region in accommodating both SEMG1 and EP055, as demonstrated by the participation of Asp71 in several interactions. Our study suggests that the SEMG1 residue Gln235 interacts with the EPPIN's Asn repeat, likely stabilizing the complex. Finally, we identified the residue Cys239 of SEMG1 interacting mainly with the WFDC domain (Glu41, Phe42, and Lys68) (Supplementary Table S3) but not with the Asn repeat, as suggested previously^24^.

Our results are also in agreement with studies demonstrating that the deletion of the EPPIN C-terminal sequence containing the Asn repeat impaired its binding to SEMG1^25^. Moreover, the anti-EPPIN S21C antibody mapping the sequence Ser103-Cys123 blocks the SEMG1 binding pocket^22,36^. Experimental assays demonstrated that EP055 and EP012 compete with SEMG1 for EPPIN binding, indicating their binding sites overlap^18^. Our MD simulations further highlight the hinge residue Asp71, which forms polar contacts during the simulation to assure the stabilization of the EPPIN complexes with SEMG1-E2Q, EP055, and EP012, revealing its relevance for the binding pocket. This hypothesis is supported by the high conservation of the hinge sequence (Asp71-Asp75) among mammalian species, including humans, non-human primates, and rodents (Supplementary Fig. S13). Taken together, it is likely that EPPIN has a druggable region formed by a tripartite interface comprising the WFDC domain, Kunitz domain, and hinge loop shaping a single binding site. This site can accommodate endogenous (SEMG1) or exogenous (EP055 or EP012) ligands, justifying their competition for EPPIN binding^18^ while highlighting the relevance of all EPPIN domains to sperm motility and function^20,22^.

Competition and functional assays demonstrated that EP012 is at least ~ 20-fold more potent in disrupting EPPIN interaction with the anti-EPPIN antibody S21C and ~ sevenfold more potent in inhibiting human sperm motility than EP055^18^. These results suggest a higher binding affinity of EP012 than EP055, which could be attributed to structural differences between EP012/EPPIN and EP055/EPPIN complexes. Although both complexes showed similar free energies, MD simulations indicated that EP012 interacts with more residues in the hinge domain (EP055: Asp71; EP012: Asp71, Gly74, and Asp75) and the Asn repeat (EP055: Asn113, Asn114; EP012: Asn113, Asn114, and Asn115) of the Kunitz domain (Supplementary Fig. S14 and Supplementary Table S4), suggesting a better fit of EP012 in the binding pocket. However, these results do not show a clear difference in the interaction pattern between the two complexes, warranting further analysis to support this hypothesis.

Although experimental data indicates that EPPIN interacts with the sperm membrane ^13,14,21^, no structural evidence has been observed to support such a hypothesis until now. Our results identified residues in both WFDC (Pro22, Thr25, Trp27, Leu28, Leu29, and Arg52) and Kunitz (Lys97, Lys98, Asn100, and Lys130, Phe132, and Pro133) domains that were important for membrane interaction and anchoring. Hydrophobic N-terminal and C-terminal residues strongly interact with phospholipid tails during MD simulations, highlighting their relevance for membrane anchoring. It is worth noting that such hydrophobic residues are found as loop regions of EPPIN, which could facilitate their penetration and stabilization into the membrane. In addition, we identified polar and positively charged residues as important for phospholipids' heads interaction, thus further stabilizing EPPIN in the cell membrane. We propose that EPPIN hydrophobic and polar residues are arranged strategically to allow membrane anchoring while maintaining its binding site exposed to the solvent to interact with endogenous or exogenous ligands. Thus, the effects of interactors, such as SEMG1, EP055, and EP012, on sperm motility may result from the stabilization of EPPIN conformation on the sperm membrane, thus leading to changes in ion influx. Consistently, it has been demonstrated that both SEMG1 and anti-EPPIN antibodies lead to intracellular Ca^2+^ and internal pH decrease in human spermatozoa^19,35^. Additional experiments are warranted to test this hypothesis.

EPPIN's novel mechanism of action and druggable properties make it an ideal sperm target for male contraception^11,22^. Our study indicates that EPPIN residues, differentially distributed in the WFDC and Kunitz domains, as well as in the hinge region, play key roles in its interaction with membranes, protein interactors, and synthetic ligands. These results may aid the rational design of more selective drug-like EPPIN ligands with potent sperm motility inhibitory activity. Considering the urgent demand for novel male contraceptives, our data may provide a new path for drug development programs for non-hormonal male contraception targeting sperm function.

## Materials and methods

### Homology modeling and conformational exploration of the full-length EPPIN model

The full-length EPPIN model (UniProtKB—O95925) was generated by homology molecular modeling of the WFDC (N-terminal—Pro22-Leu70) and Kunitz (C-terminal—Val76-Pro133) domains, which were connected by the hinge segment (Asp71–Asp75). For that, different experimental structures were used as templates (PDB IDs: 2REL^37^, 1FLE^38^, 2Z7F^39^, 1ADZ^40^, and 1BIK^41^, which were aligned by HHPRED and further submitted to Modeller v9.12^42,43^. The best out of 100 models was selected based on the DOPE score. The structural quality of the model was validated using the MolProbity server v4.2^44^.

MD simulations were performed to explore the conformational space of the EPPIN model, using GROMACS v5.0.5 or v2019.1^45^ under the GROMOS96 54a7 force field^46^. Residue protonation was set at a neutral pH according to the PROPKA3 web server^47^. The model was placed in a cubic box 12 Å distant from the farthest atom in XYZ directions, which was further solvated, neutralized, and equilibrated with 0.15 M NaCl. The system was submitted to a minimization step using the Steepest Descent algorithm until reaching an energy gradient below 100 kJ/mol/nm^2^. Initial velocities were generated randomly to a Maxwell–Boltzmann distribution at 310 K, then an NVT ensemble was applied using the V-Rescale thermostat^48^ with a time constant of 0.1 ps, followed by an NPT ensemble of 1 ns, monitoring the pressure at 1 bar using the Berendsen barostat^49^ with a time constant of 1.0 ps. These two steps were performed under position restraints of EPPIN's backbone atoms with a force constant of 1000 kJ/mol/nm^2^. An unconstrained production step was performed during 500 ns using the Nose–Hoover thermostat^50,51^ and Parrinello–Rahman barostat^52^, with a time constant of 0.5 and 5.0 ps, respectively. Non-bonded van der Waals and coulomb interactions were calculated considering atoms within 14 Å using the PME method. Three independent replicas were performed, collecting frames every 10 ps.

We also submitted the initial structure of the full-length EPPIN model to Normal Mode Analysis (NMA) to identify energetically favorable conformational changes to be compared to MD simulations. These conformational changes can be captured by low-frequency normal modes that are known to be related to the function of proteins [Bahar]. To this end, all-atom elastic normal modes (aanma) were calculated using Bio3D^53^, with rotation-translation block approximation of one amino acid residue per block.

### Identification of druggable hotspots and interaction of EPPIN to SEMG1, EP055, and EP012

After exploring the conformational space of EPPIN using MD simulations, we clusterized (see the “Structural analysis” section below) frames from all three replicas to obtain central structures (Cla, Clb, and Clc) of the three most populated conformations, which were submitted to the FTMap web server to identify druggable regions. Following, these regions were considered for further docking analyses against the semenogelin-1 peptide (SEMG1-E2Q) and organic molecules (EP055 and EP012). SEMG1-E2Q peptide structure was modeled using the PEP-FOLD web server^54^, while small organic molecules were modeled using the Automatic Topology Builder (ATB)^55^.

Molecular docking assays were performed using Patchdock^26^ and DockThor servers^27^ for SEMG1-E2Q and small organic molecules, respectively. Patchdock parameters were standard, while DockThor parameters were set to determine a grid box covering the model, with a discretization, population size, number of evaluations, and number of runs set as 0.25, 1000, 1,000,000, and 20, respectively. For both SEMG1-E2Q peptide and small organic molecules, the best two poses were selected according to their binding energy score (kcal/mol) and then submitted to an MD simulation step following the previous parameters. Initially, every pose was simulated during 100 ns, and then the most stable systems were extended until 500 ns.

### Orientation of EPPIN in the cell membrane using the orientation of proteins in membranes (OPM) server

The orientation of EPPIN in the cell membrane was predicted using the OPM server, which measures the interaction of proteins or peptides to the lipid bilayer through a polarity profile based on the free energy of transfer of molecules from water to the anisotropic lipid environment using PPM 2.0 method^29^. We selected the EPPIN/SEMG1-E2Q peptide complex of the most representative conformation of EPPIN (Cla) to be placed in a POPC membrane The equilibrated membrane coordinates were obtained from a previous study ^56^, then the EPPIN/SEMG1-E2Q peptide complex was oriented according to the OPM server, removing the lipids that were clashing with the complex. Further, the system was submitted to an MD simulation step lasting 100 ns, following the same protocol as previously described.

### Structural analysis

Trajectories were processed to remove the Periodic Boundary Condition for each MD simulation set, and then frames were analyzed. Root-mean-square deviation (RMSD), radius of gyration (Rg), clusterization, Gibbs free energy landscape, Poisson-Boltzmann surface area method (MM/PBSA), and Principal Component Analysis (PCA) calculations were performed using GROMACS built-in tools.

Gibbs free energy calculation was performed using *gmx sham* tool, which converts a set of collective variables in a histogram of density distributions. Values of two collective variables, Phe63-Asn repeat distance and the interdomain angle, sampled during MD simulations were projected on a bidimensional grid, with the highest density area set to zero. Temperature and grid were set to 310 K and 120 bins, respectively. The last 1 ns of the MD simulations was selected to calculate the binding energies of the EPPIN/EP055 complex using the MM/PBSA method implemented in GROMACS^28^, selecting the solute and solvent dielectric constants values of 2 and 80, respectively. PCA calculations were performed for each replica to obtain main displacement vectors from MD simulations using the *gmx anaeig* tool, which were compared to those vectors generated from NMA. Clusterization was performed using the GROMOS method^57^ with a cutoff below 4 Å, discarding the first 50 ns of each replica.

The prevalence of contacts for each residue of EPPIN in contact with SEMG1-E2Q, EP055, or POPC was measured considering heavy atoms within a 3.5 Å cutoff, using a homemade tcl script implemented in VMD^58^. Interaction types were obtained by two-dimension diagrams of protein-peptide and protein–ligand complex using Discovery Studio Visualizer 21.1.0.20298. Water molecules occupancy was calculated in a resolution of 1 Å and isovalue of 0.44, using the built-in tool VolMap available in VMD^58^. All structural graphics were produced with VMD^58^, and all plots were built using gnuplot v5.2 (http://www.gnuplot.info).

### Supplementary Information

Supplementary Information.

Supplementary Video S1.

## Data Availability

All relevant data and their Supporting Information are within the manuscript. Examples of the scripts used in the prevalent contact evaluation and minimal distance analysis are available at: https://github.com/antonielgomes/EPPIN.git.
