# Supplementary material for: Interactions of the male contraceptive target EPPIN with semenogelin-1 and small organic ligands
Source: Sci Rep. 2023 Sep 1;13:14382. doi: 10.1038/s41598-023-41365-1 (PMC10474283; doi:10.1038/s41598-023-41365-1)
Supplement: Supplementary file 1 — Supplementary Information. [file 41598_2023_41365_MOESM1_ESM.pdf]

Supplementary Materials for

Interactions of the male contraceptive target EPPIN with semenogelin-1 and small organic ligands.

Antoniel A.S. Gomes[1,2#*], Natália C.M. Santos[1], Leonardo R. Rosa[1], Rafael J. Borges[1,3], Marcos R.M. Fontes[1,4], Katherine G. Hamil[5], Michael G. O'Rand[5,6], Erick J.R. Silva[1*].

[1] Department of Biophysics & Pharmacology, Institute of Biosciences of Botucatu, São Paulo State University, Botucatu-SP, Brazil.

[2] Laboratory of Biological Physics, Carlos Chagas Filho Institute of Biophysics, Universidade Federal do Rio de Janeiro, Rio de Janeiro-RJ, Brazil.

[3] The Center of Medicinal Chemistry (CQMED), Center for Molecular Biology and Genetic Engineering (CBMEG), University of Campinas (UNICAMP), Campinas, Brazil

4 Institute for Advanced Studies of the Sea (IEAMAR), São Paulo State University, UNESP, São Vicente, SP, Brazil

[5] Research and Development, Eppin Pharma Inc., Chapel Hill, North Carolina, United States of America.

[6] Department of Cell Biology & Physiology, University of North Carolina at Chapel Hill, Chapel Hill, North Carolina, United States of America.

[#] Current Address:

* Corresponding authors

E-mails:

antonielaugusto@gmail.com (AASG)

ejr.silva@unesp.br (EJRS)

Supplementary Figures

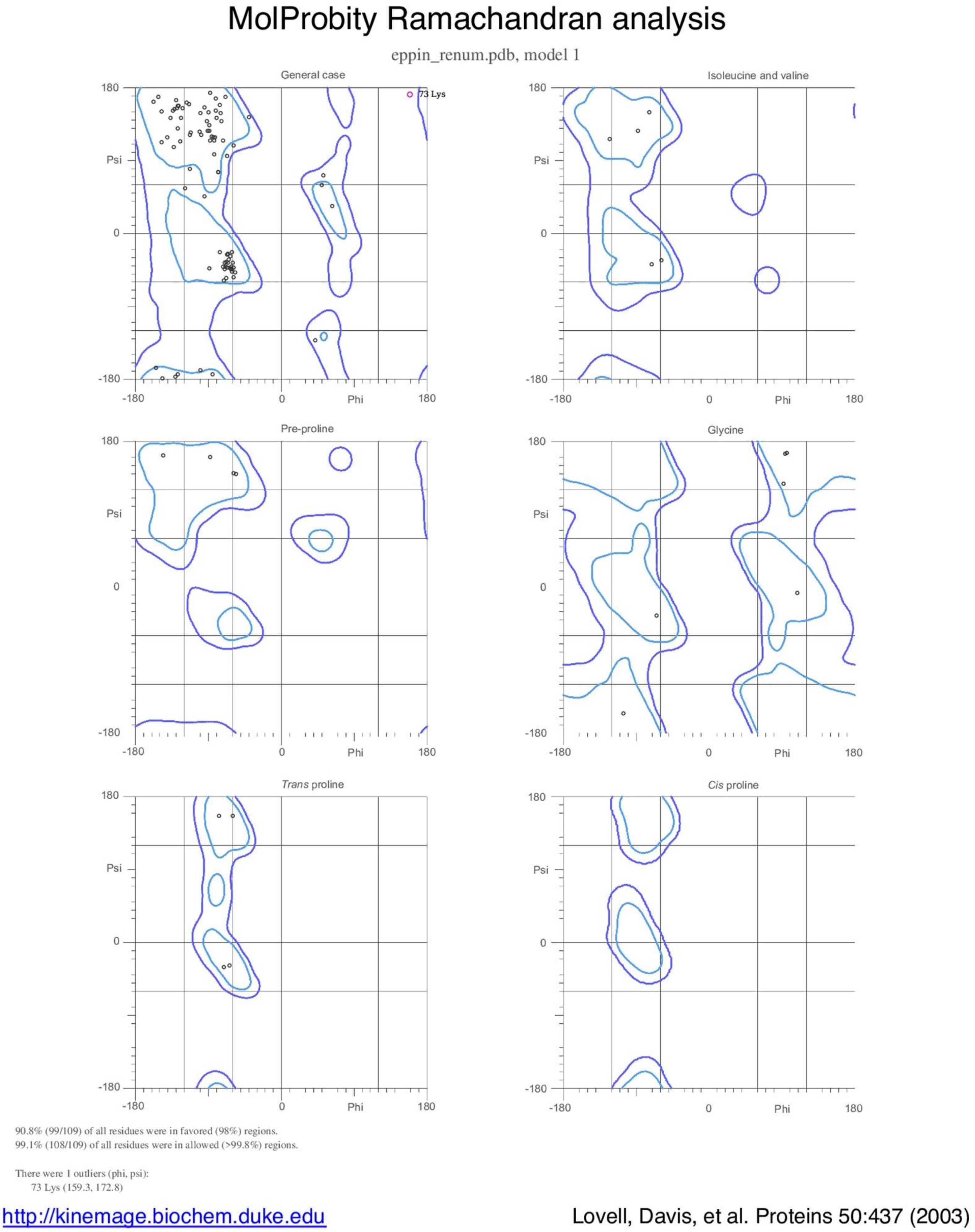

Supplementary Fig. S1. Ramachandran plot of the generated EPPIN model. Residues in allowed regions are shown as black circles, while outliers are shown as red circles. The representation shows 99.1% of all residues located in allowed regions.

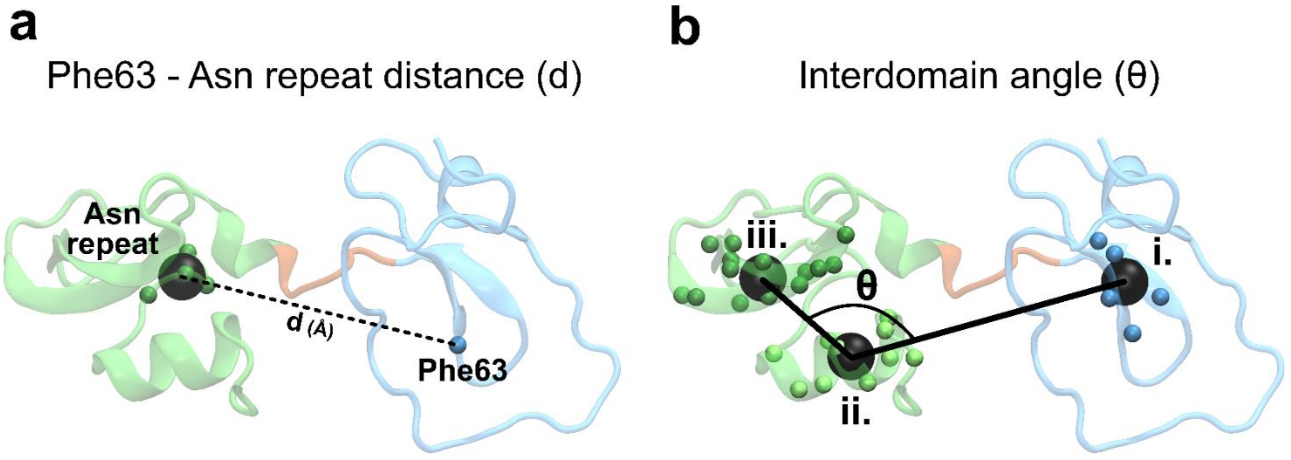

Supplementary Fig. S2. Reaction coordinates defined to evaluate EPPIN conformations. (a) The Phe63-Asn repeat distance measures the distance of Phe63's Cα atom and the center of mass of Cα atoms of the Asn repeat (Asn113-Asn116). (b) The interdomain angle (θ) was obtained by determining the center of mass of Cα atoms (black sphere) in three regions of EPPIN's model: i. Cys60-Val62 and Lys67-Cys69 (cyan sphere); ii. Lys120-Asn129 (light green sphere); and iii. Phe90-Asp96 and Asn100-Tyr107 (dark green sphere). EPPIN is shown as cartoon in cyan, green, and brown for the WFDC domain, the Kunitz domain, and the hinge, respectively.

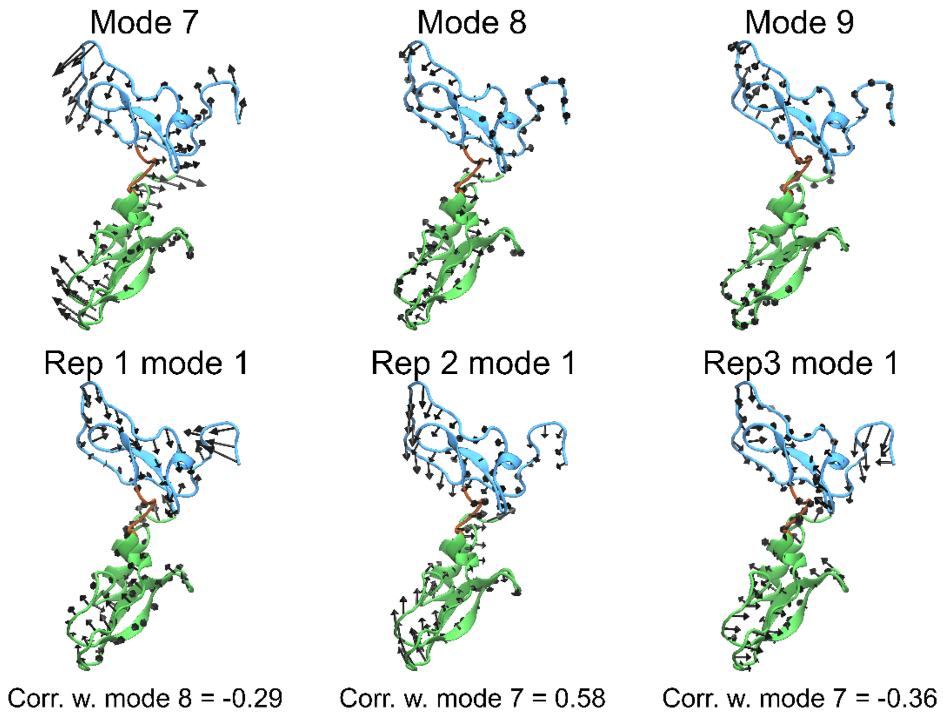

Supplementary Fig. S3. Main global motions of the full-length EPPIN model by normal mode analysis (MNA) and MD simulations. Motions of the three lowest-frequency modes (black arrows) at the top. Replica correlation between the main MD eigenvectors and the best mode (Corr. w. mode) at the bottom.

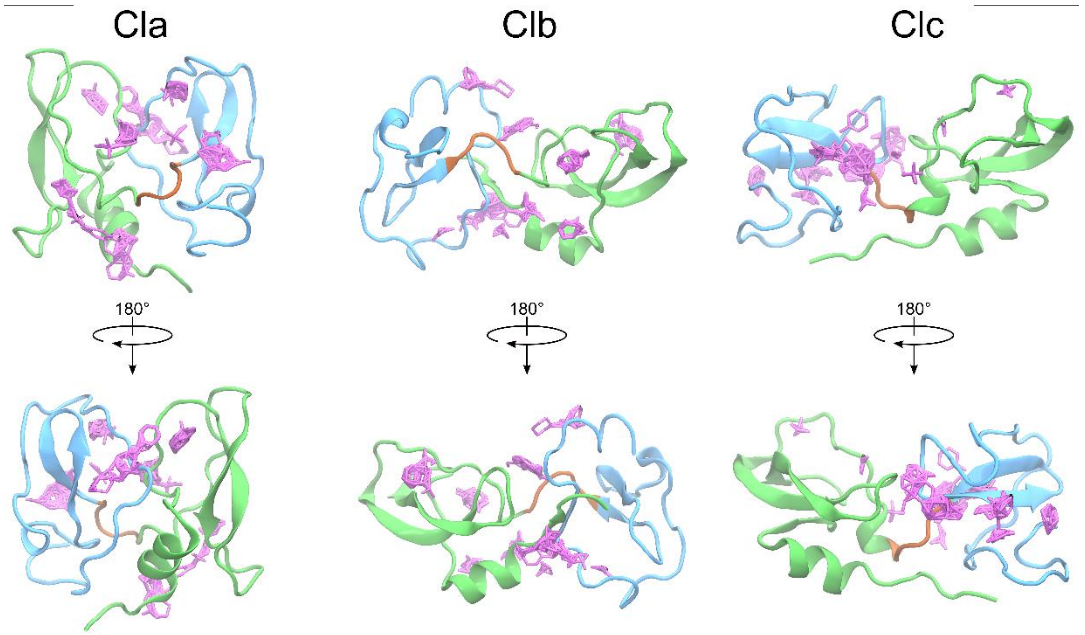

Supplementary Fig. S4 FTMap of the three central cluster conformation (Cla, Clb, and Clc) of the EPPIN model with different possible druggable regions. The upper and lower panel shows different positions of EP055 in front and back of EPPIN for each state conformation, respectively. EPPIN WFDC domain (blue), Kunitz domain (green) and hinge segment (brown), and EP055 ligand (purple) are shown. Docking scores were obtained from Patchdock, shown as S in parentheses.

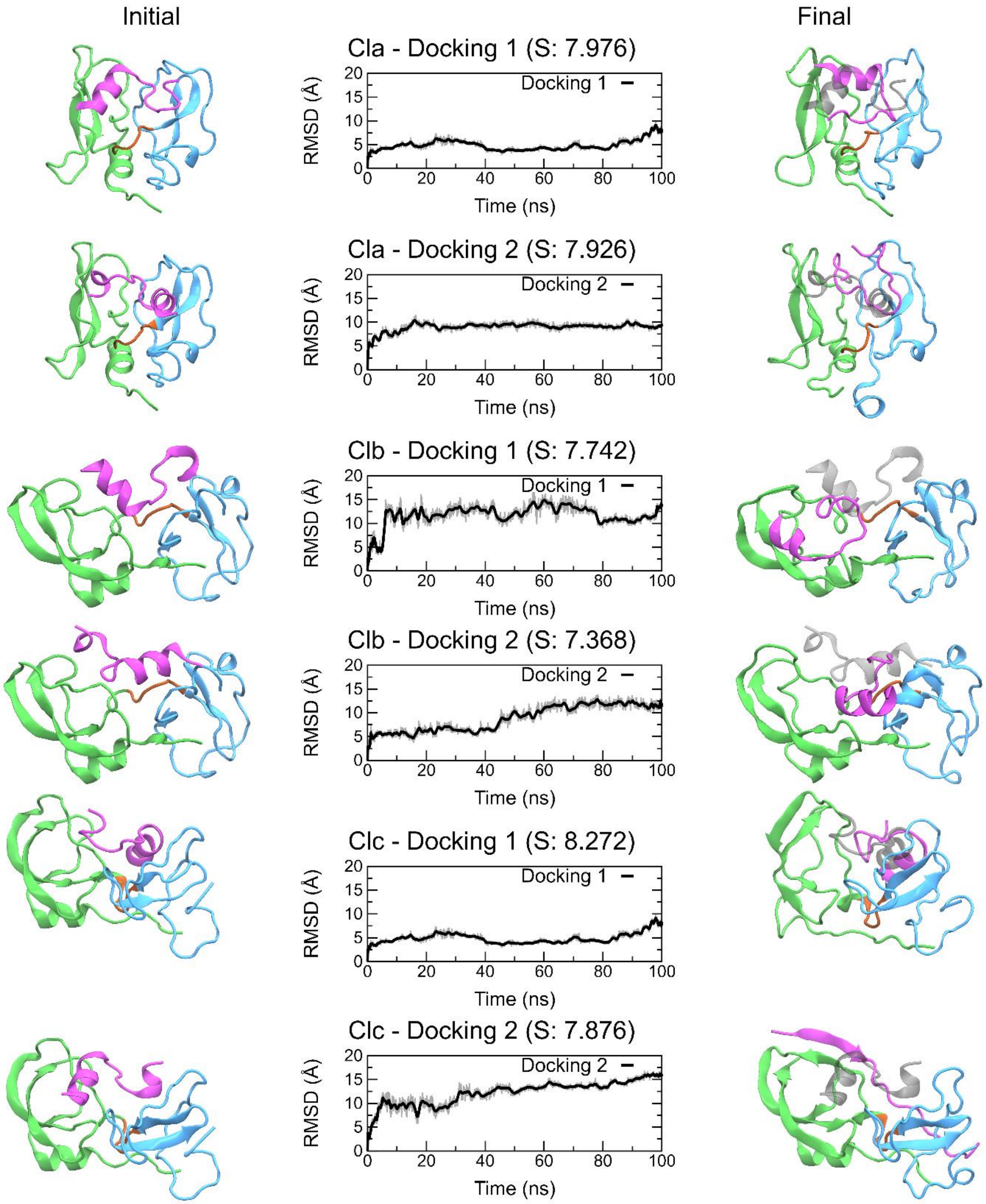

Supplementary Fig. S5. Temporal RMSD of SEMG1 backbone atoms interacting with the full-length EPPIN model during the 100 ns MD simulation. EPPIN WFDC domain (blue), Kunitz domain (green), and hinge segment (brown) are shown. The SEMG1-E2Q peptide positions are shown as the initial in purple and the final orientation, with the latter showing the superposition onto the initial (grey) and after 100 ns (purple). Docking scores were obtained from Patchdock, shown as S in parentheses.

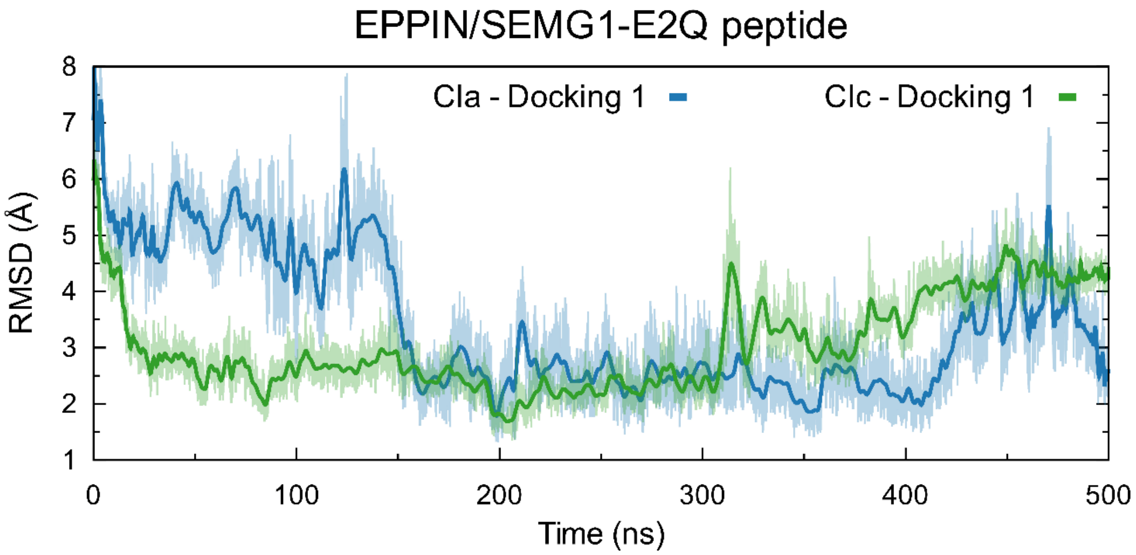

Supplementary Fig. S6. Temporal RMSD of the SEMG1-E2Q peptide backbone atoms in comparison with the average conformation of the complex EPPIN/SEMG1. Docking 1 of Cla and Clc are shown in blue and green, respectively.

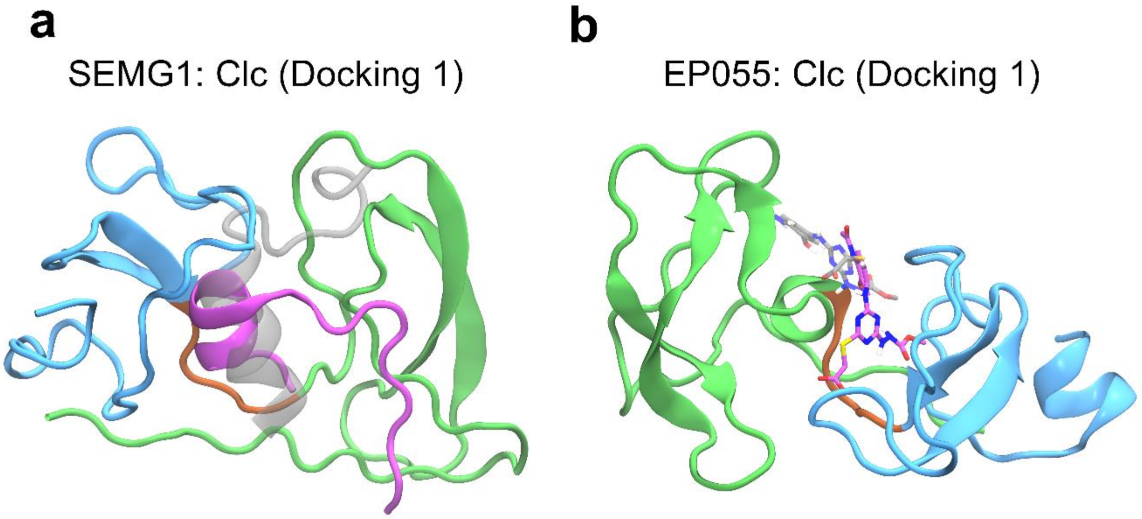

Supplementary Fig. S7. Orientations of Docking 1 of Clc for the (a) EPPIN/SEMG1 and (b) EPPIN/EP055 complexes during the 100 ns MD simulation. Initial and final conformations of SEMG1 and EP055 are shown in gray and purple, respectively. EPPIN WFDC domain (blue), Kunitz domain (green), and hinge segment (brown) are shown.

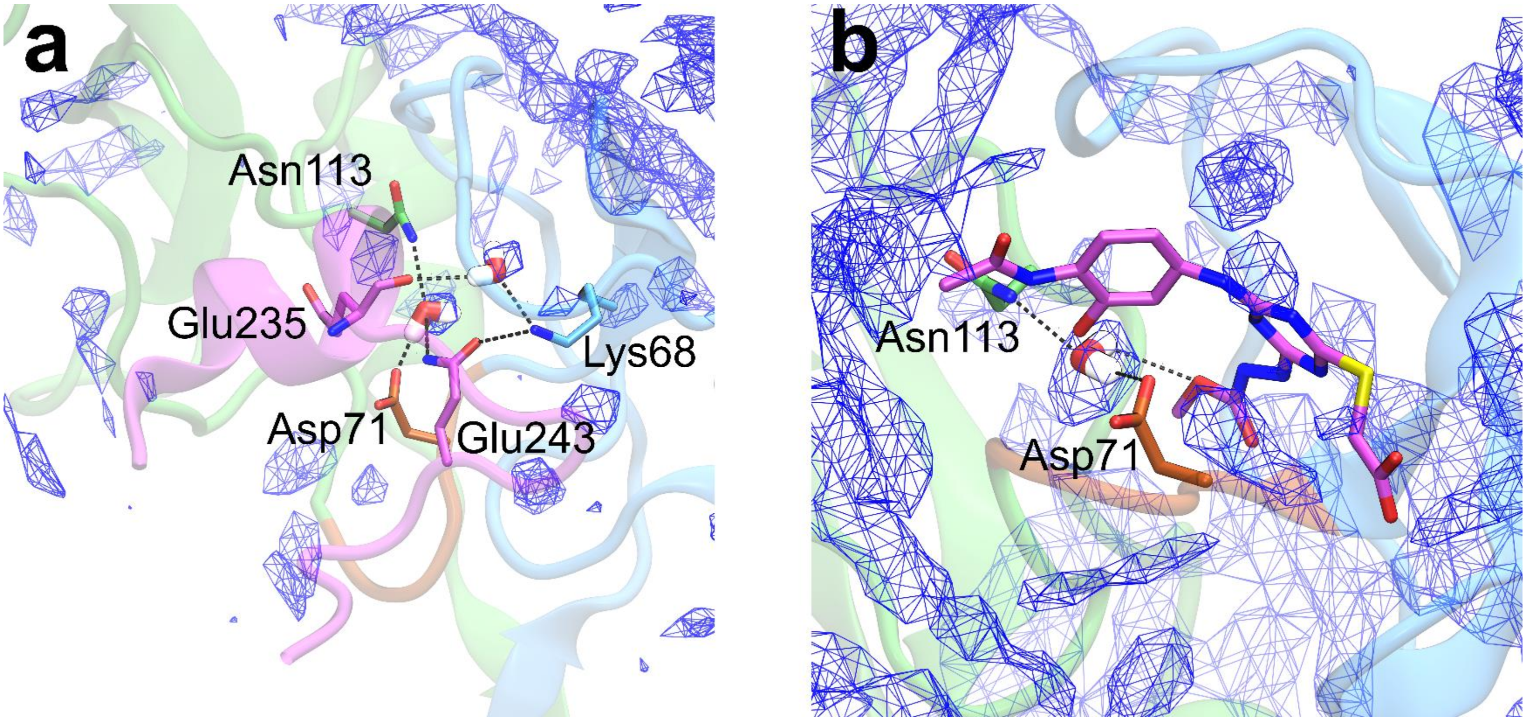

Supplementary Fig. S8. Identification of water molecules in the interface of the (a) EPPIN/SEMG1 and (b) EPPIN/EP055 complexes. EPPIN residues are shown in cyan, brown, and green for the WFDC, hinge, and Kunitz domains, respectively. SEMG1 or EP055 are shown as purple. Important residues are highlighted as sticks. Hydrogen bonds under 3 A are shown as black dotted lines. Water occupancy is shown as blue meshes.

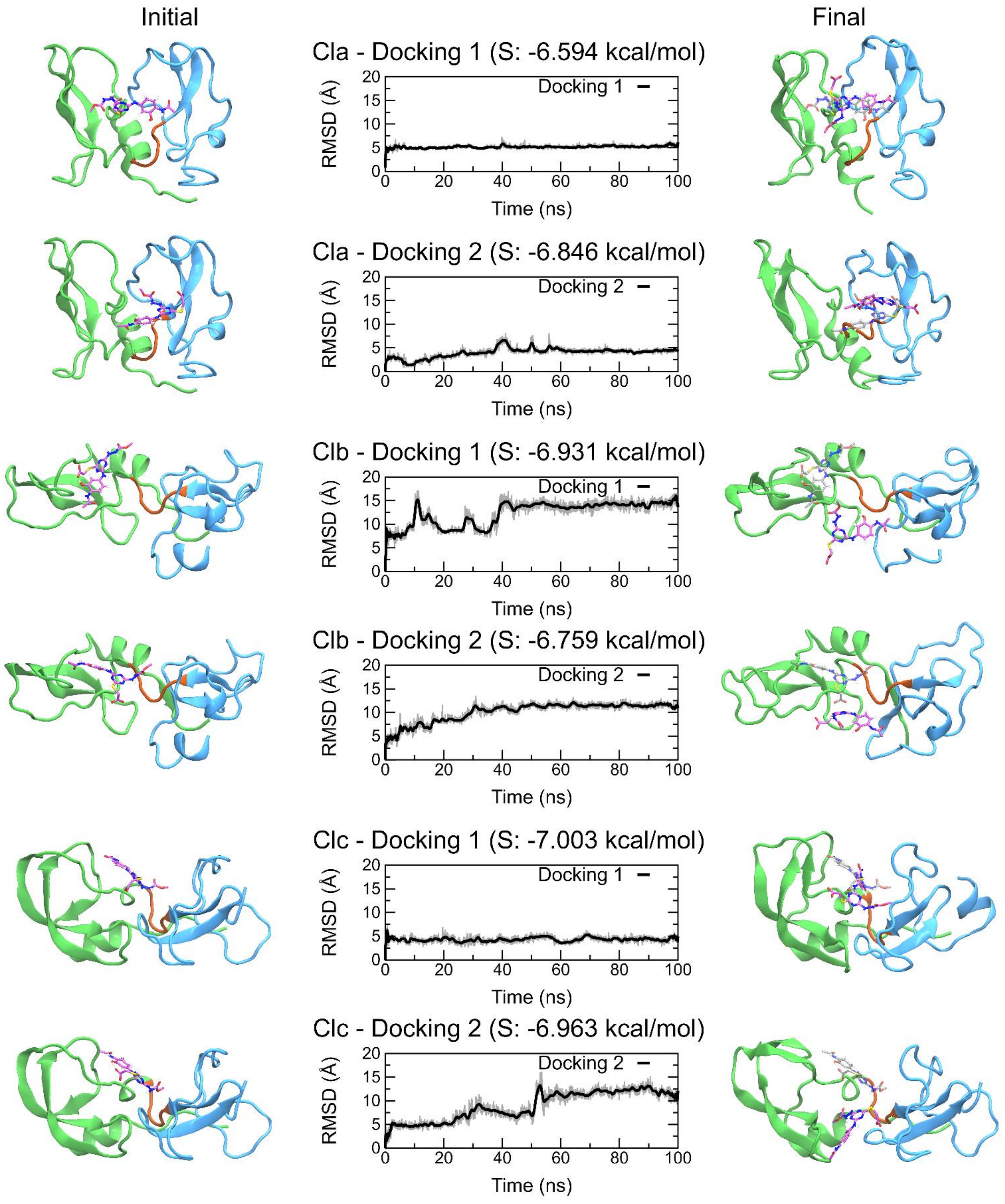

Supplementary Fig. S9. Temporal RMSD of EP055 heavy atoms interacting with the full-length EPPIN model during 100 ns MD simulation. EPPIN WFDC domain (blue), Kunitz domain (green), and hinge segment (brown) are shown. The EP055 positions are shown as the initial purple and final orientations, with the latter showing the superposition onto the initial (grey) and after 100 ns (purple). Docking scores were obtained from DockThor, shown as S in parentheses.

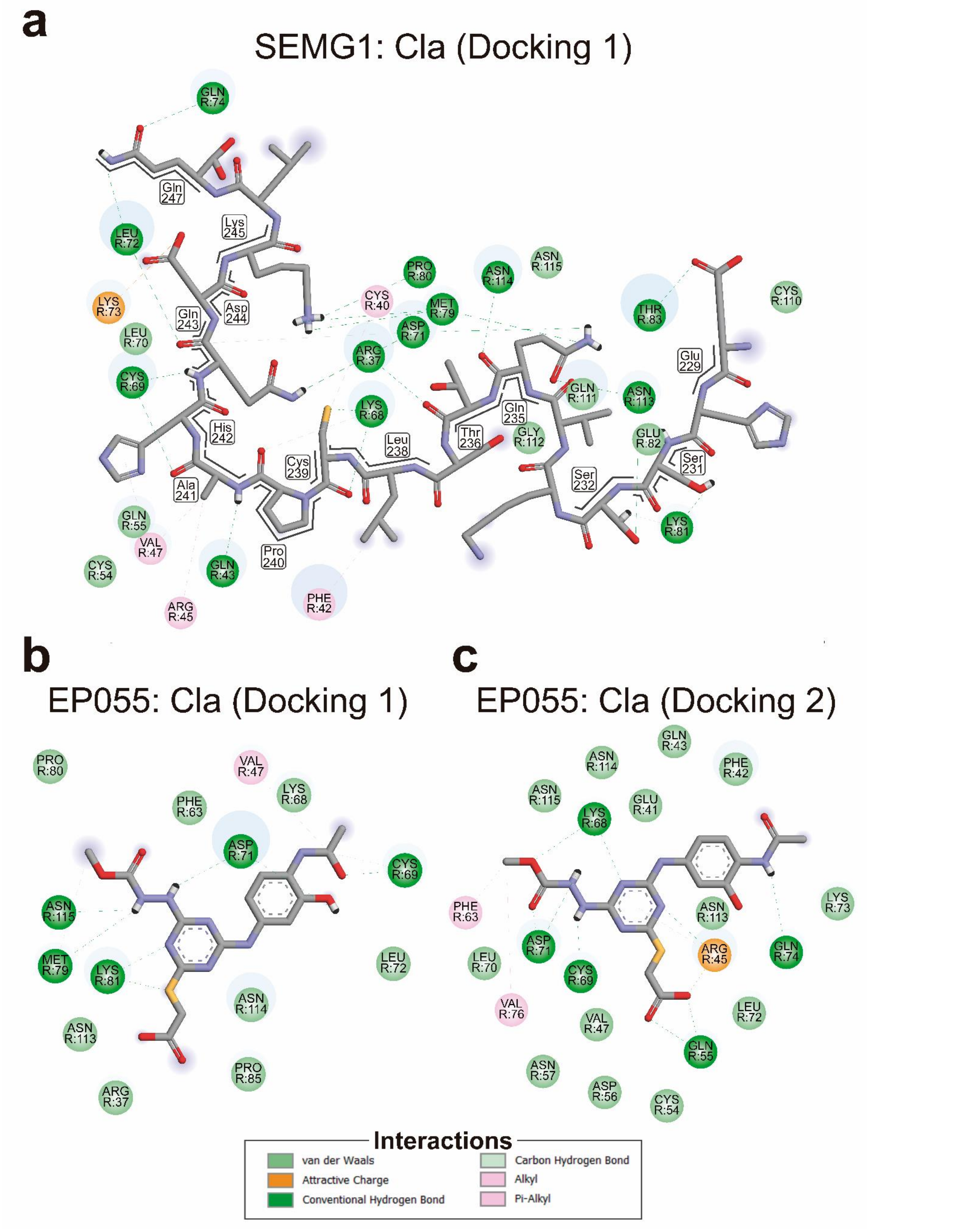

Supplementary Fig. S10. Diagrams illustrating the types of molecular interactions between EPPIN and (a) SEMG1 or (b and c) EP055. Molecular interactions are demonstrated as hydrogen bonds (dark green), Van der Waals bonds (light green), alkyl halides (purple), and attractive charge (orange). EPPIN residues are shown inside of circles, and ligands are shown as 2D representations. SEMG1 residues are highlighted as gray boxes.

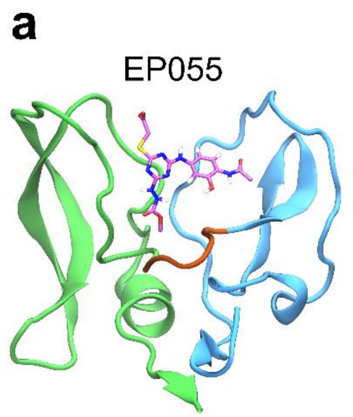

Supplementary Fig. S11. Interaction between the full-length EPPIN model with EP055, EP012, and SEMG1-E2Q peptide. (a) EP055 is shown in purple, (b) EP012 is shown in purple, with EP055 shown in gray, and (c) SEMG1-E2Q peptide shown in purple, with EP055 shown in gray.

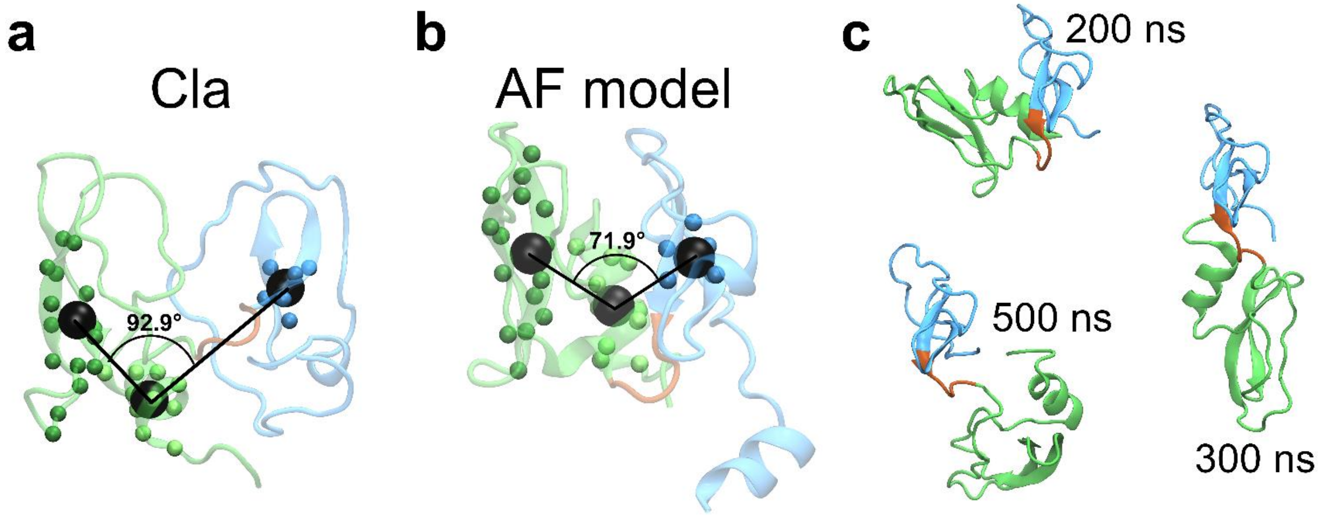

Supplementary Fig. S12. Interdomain angle calculated for different EPPIN models. (a) Value for the EPPIN model in the most visited conformation (Cla), (b) for the EPPIN model predicted by AlphaFold (AF), and (c) different conformations of the AF model obtained after 200, 300, and 500 ns of MD simulation.

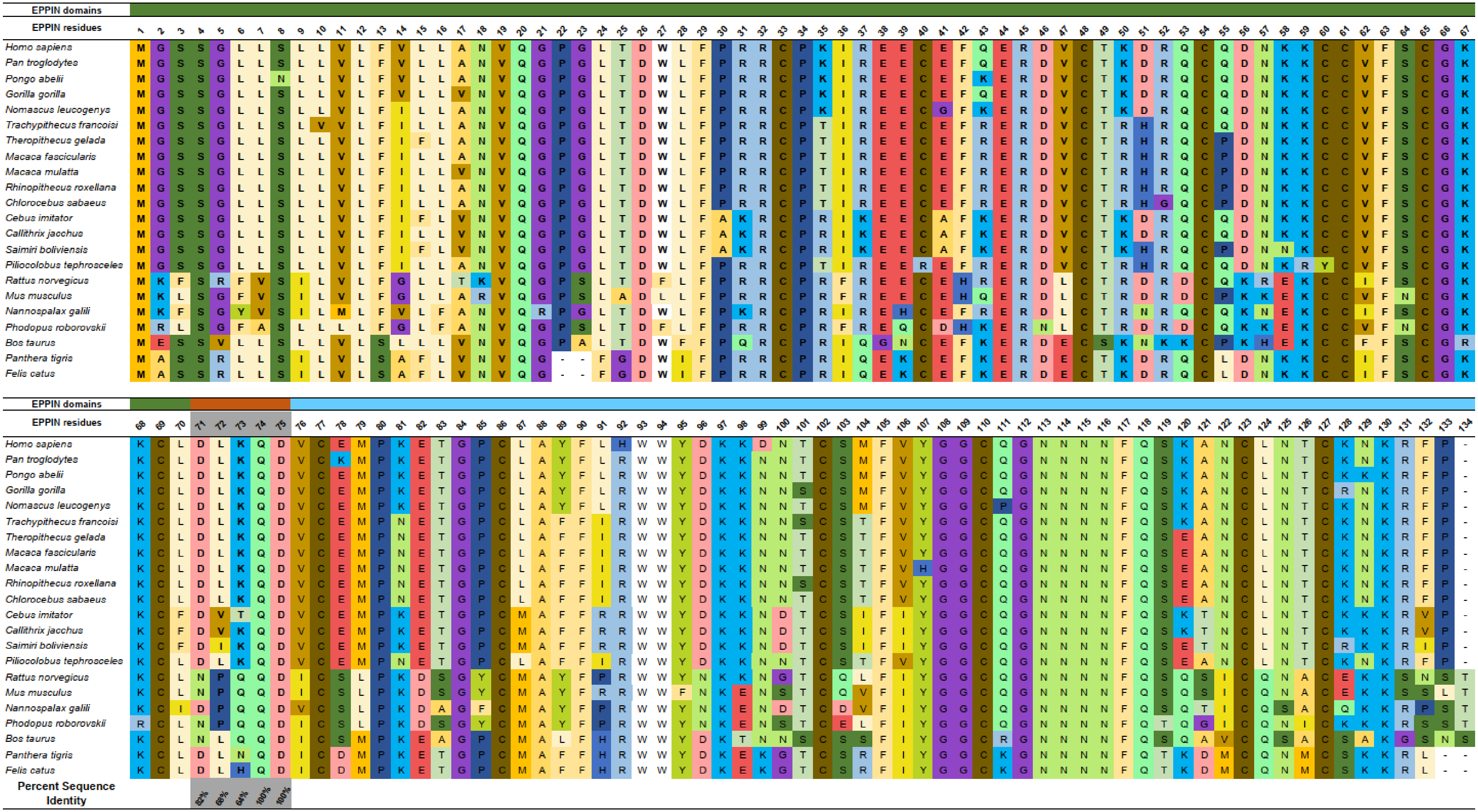

Supplementary Fig. S13. Alignment of EPPIN sequences from different mammalian species (primates, rodents, felines, and bovine) evidencing the high conservation of the hinge segment (Asp71-Asp75, in bold). Species and EPPIN accession numbers are indicated: *Homo sapiens* (NP 065131.1); *Pan troglodytes* (XP 003317013.1); *Pongo abelii* (XP 009231918.1); *Gorilla gorilla* (XP 004062305.1); *Nomascus leucogenys* (XP 003253690.1); *Trachypithecus francoisi* (XP 033042444.1); *Theropithecus gelada* (XP 025230353.1); *Macaca fascicularis* (XP 005569206.3); *Macaca mulatta* (NP 001028013.1); *Rhinopithecus roxellana* (XP 010382527.1); *Chlorocebus sabaeus* (XP 037838657.1); *Cebus imitator* (XP 017396723.1); *Callithrix jacchus* (XP 008994257.1); *Saimiri boliviensis* (XP 003936493.1); *Piliocolobus tephrosceles* (XP 026308201.1); *Rattus norvegicus* (NP 001102927.1); *Mus musculus* (NP 083601.1); *Nannospalax galili* (XP 017650696.1); *Phodopus roborovskii* (CAH6776815.1); *Bos taurus* (NP 001070530.1); *Panthera tigris* (XP 007077828.1); *Felis catus* (XP 006929779.1).

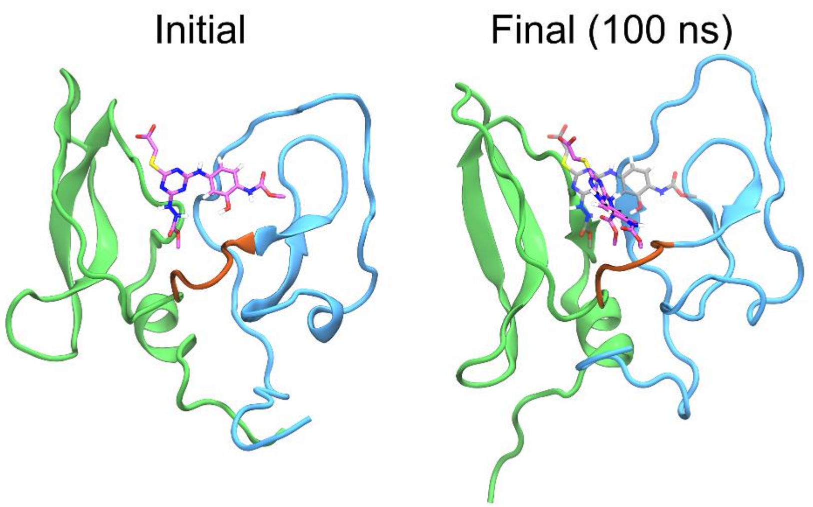

Supplementary Fig. S14. Interaction between the full-length EPPIN model with EP012 during the 100 ns MD simulation. EPPIN WFDC domain (blue), Kunitz domain (green), and hinge segment (brown) are shown. The EP012 positions are shown as initial (purple; left panel) and final, showing the superimpose onto the initial (grey, right panel) and the final conformation 100 ns (purple, right panel).

Supplementary Tables

Supplementary Table S1. Parameters used for the determination of full-length human EPPIN (without the signal peptide) three-dimensional models using the structure of proteins containing WFDC-type and Kunitz-type protease inhibitor domains as templates.

Template (PDB ID)	Protease inhibitor domain	Sequence Identity (%)	Coverage (%)
2Z7F	WFDC	34.09	40.18
2REL	WFDC	32.69	47.32
1FLE	WFDC	41.46	37.50
1ADZ	Kunitz	35.59	52.68
1BIK	Kunitz	48.21	48.21

Supplementary Table S2. Intermolecular H-bonds (≤3.5 Ä) in EPPIN/SEMG1-E2Q complexes during molecular dynamic simulations.

% Interaction overtime (500 ns)		
**EPPIN residue**	**EPPIN/SEMG1-E2Q complex**	
	**Cla Docking 1**	**Clc Docking 1**
P22	9.01	0.03
G23	5.14	0.0
L24	0.52	1.52
T25	0.15	0.00
D26	0.0	0.0
W27	1.67	0.0
K28	0.0	0.0
F29	0.0	0.0
P30	0.0	0.0
R31	0.0	0.0
R32	0.0	0.0
C33	0.0	0.0
P34	0.0	2.71
K35	0.0	0.30
I36	0.06	31.35
R37	68.5	3.18
E38	0.04	89.19
E39	26.0	37.06
C40	22.4	54.77
E41	92.9	98.76
F42	84.1	0.00
Q43	78.7	66.47
E44	0.62	98.69
R45	24.9	7.24
D46	0.92	1.57
V47	27.4	7.22
C48	0.0	0.05
T49	0.0	0.00

K50	0.0	0.0
D51	0.0	0.0
R52	0.0	0.0
Q53	3.40	0.0
C54	1.89	0.0
Q55	46.1	0.24
D56	7.71	0.27
N57	0.01	9.82
K58	0.0	0.0
K59	0.0	0.0
C60	0.0	0.0
C61	0.0	0.0
V62	0.0	0.0
F63	37.1	72.32
S64	0.0	0.0
C65	0.00	0.02
G66	0.0	0.0
K67	0.0	0.06
K68	92.0	97.67
C69	54.3	15.52
L70	2.42	21.00
D71	94.5	14.77
L72	89.0	0.02
K73	63.6	9.94
Q74	68.5	54.99
D75	1.75	87.33
V76	0.00	26.75
C77	0.0	5.51
E78	5.14	56.64
M79	77.9	32.86
P80	36.8	52.56
K81	15.0	52.42
E82	50.2	48.57
T83	21.6	28.27
G84	0.26	0.01
P85	0.0	0.01
C86	0.04	0.00
L87	0.0	0.00
A88	0.0	0.0
Y89	0.0	0.0
F90	0.0	9.91
L91	0.0	0.30
H92	0.0	6.71
W93	0.0	0.02
W94	0.00	2.25
Y95	0.0	0.0
D96	0.0	0.02
K97	0.01	0.60
K98	0.0	0.04
D99	0.0	0.0

N100	0.0	0.0
T101	0.0	0.0
C102	0.0	0.0
S103	0.0	0.0
M104	0.0	0.0
P105	0.0	0.0
V106	0.0	0.0
Y107	0.28	5.97
G108	0.0	0.0
G109	0.0	0.0
C110	0.51	0.33
Q111	69.1	72.56
G112	57.1	57.19
N113	83.8	68.92
N114	98.0	70.74
N115	80.7	56.59
N116	0.0	26.80
F117	0.0	25.45
Q118	0.0	23.46
S119	0.0	21.48
K120	0.0	22.28
A121	0.0	0.0
N122	0.0	5.57
C123	0.0	5.53
L124	0.0	16.99
N125	0.0	14.56
T126	0.0	3.40
C127	0.0	0.31
K128	0.0	0.54
N129	0.0	0.0
K130	0.0	80.39
R131	0.94	0.00
F132	0.0	0.01
P133	0.0	0.0

Supplementary Table S3. Intermolecular H-bonds (≤3.5 Ä) in EPPIN/SEMG1-E2Q peptide complex during molecular dynamic simulations.

% Interaction overtime (500 ns)																			
**EPPIN**	**SEMG1 peptide residue**																		
	E229	H230	S231	S232	K233	V234	Q235	T236	S237	L238	C239	P240	A241	H242	Q243	D244	K245	L246	Q247
P22	0.0	0.0	0.0	0.0	0.0	0.0	0.0	0.0	0.0	0.0	0.0	0.0	0.0	0.8	0.8	2.2	0.2	0.0	8.4
G23	0.0	0.0	0.0	0.0	0.0	0.0	0.0	0.0	0.0	0.0	0.0	0.0	0.0	0.0	0.0	0.1	0.0	0.1	5.1
L24	0.0	0.0	0.0	0.0	0.0	0.0	0.0	0.0	0.0	0.0	0.0	0.0	0.0	0.0	0.0	0.0	0.0	0.2	0.5
T25	0.0	0.0	0.0	0.0	0.0	0.0	0.0	0.0	0.0	0.0	0.0	0.0	0.0	0.0	0.0	0.0	0.0	0.0	0.2
D26	0.0	0.0	0.0	0.0	0.0	0.0	0.0	0.0	0.0	0.0	0.0	0.0	0.0	0.0	0.0	0.0	0.0	0.0	0.0
W27	0.0	0.0	0.0	0.0	0.0	0.0	0.0	0.0	0.0	0.0	0.0	0.0	0.0	0.0	0.0	0.0	0.0	0.0	2.9
K28	0.0	0.0	0.0	0.0	0.0	0.0	0.0	0.0	0.0	0.0	0.0	0.0	0.0	0.0	0.0	0.0	0.0	0.0	0.0
F29	0.0	0.0	0.0	0.0	0.0	0.0	0.0	0.0	0.0	0.0	0.0	0.0	0.0	0.0	0.0	0.0	0.0	0.0	0.0
P30	0.0	0.0	0.0	0.0	0.0	0.0	0.0	0.0	0.0	0.0	0.0	0.0	0.0	0.0	0.0	0.0	0.0	0.0	0.0
R31	0.0	0.0	0.0	0.0	0.0	0.0	0.0	0.0	0.0	0.0	0.0	0.0	0.0	0.0	0.0	0.0	0.0	0.0	0.0
R32	0.0	0.0	0.0	0.0	0.0	0.0	0.0	0.0	0.0	0.0	0.0	0.0	0.0	0.0	0.0	0.0	0.0	0.0	0.0
C33	0.0	0.0	0.0	0.0	0.0	0.0	0.0	0.0	0.0	0.0	0.0	0.0	0.0	0.0	0.0	0.0	0.0	0.0	0.0
P34	0.0	0.0	0.0	0.0	0.0	0.0	0.0	0.0	0.0	0.0	0.0	0.0	0.0	0.0	0.0	0.0	0.0	0.0	0.0
K35	0.0	0.0	0.0	0.0	0.0	0.0	0.0	0.0	0.0	0.0	0.0	0.0	0.0	0.0	0.0	0.0	0.0	0.0	0.0
I36	0.0	0.0	0.0	0.0	0.0	0.0	0.1	0.0	0.0	0.0	0.0	0.0	0.0	0.0	0.0	0.0	0.0	0.0	0.0
R37	0.0	0.0	0.1	5.4	0.1	0.6	13.0	67.7	13.9	2.2	11.3	0.3	0.0	0.0	0.0	0.0	0.0	0.0	0.0
E38	0.0	0.0	0.0	0.0	0.0	0.0	0.0	0.0	0.0	0.0	0.0	0.0	0.0	0.0	0.0	0.0	0.0	0.0	0.0
E39	0.0	0.0	0.0	0.0	0.5	0.0	0.0	23.6	3.1	0.0	1.6	0.0	0.0	0.0	0.0	0.0	0.0	0.0	0.0
C40	0.0	0.0	0.0	0.0	0.0	0.0	0.0	15.8	0.4	0.0	7.5	0.1	0.0	0.0	0.0	0.0	0.0	0.0	0.0
E41	0.0	0.0	0.0	0.0	0.5	0.0	0.0	64.2	60.6	12.7	79.6	0.0	0.0	0.0	0.0	0.0	0.0	0.0	5.0
F42	0.0	0.0	0.0	0.0	0.2	0.0	0.0	0.1	27.0	48.1	75.2	25.6	1.6	0.0	0.0	0.0	0.0	2.8	6.0
Q43	0.0	0.0	0.0	0.0	0.0	0.0	0.0	0.0	1.7	1.7	56.7	23.8	64.2	0.4	0.0	0.0	0.2	1.1	5.3
E44	0.0	0.0	0.0	0.0	0.0	0.0	0.0	0.0	0.0	0.0	0.0	0.0	0.0	0.0	0.0	0.0	0.0	0.0	0.7
R45	0.0	0.0	0.0	0.0	0.0	0.0	0.0	0.0	0.9	2.0	8.6	2.0	14.7	4.0	0.1	0.0	0.2	0.3	5.5
D46	0.0	0.0	0.0	0.0	0.0	0.0	0.0	0.0	0.0	0.0	0.0	0.0	0.0	1.6	0.0	0.0	0.0	0.0	0.0
V47	0.0	0.0	0.0	0.0	0.0	0.0	0.0	0.0	0.0	0.0	0.4	0.4	17.6	38.9	1.9	0.0	0.0	0.1	0.0
C48	0.0	0.0	0.0	0.0	0.0	0.0	0.0	0.0	0.0	0.0	0.0	0.0	0.0	0.0	0.0	0.0	0.0	0.0	0.0
T49	0.0	0.0	0.0	0.0	0.0	0.0	0.0	0.0	0.0	0.0	0.0	0.0	0.0	0.0	0.0	0.0	0.0	0.0	0.0
K50	0.0	0.0	0.0	0.0	0.0	0.0	0.0	0.0	0.0	0.0	0.0	0.0	0.0	0.0	0.0	0.0	0.0	0.0	0.0
D51	0.0	0.0	0.0	0.0	0.0	0.0	0.0	0.0	0.0	0.0	0.0	0.0	0.0	0.0	0.0	0.0	0.0	0.0	0.0
R52	0.0	0.0	0.0	0.0	0.0	0.0	0.0	0.0	0.0	0.0	0.0	0.0	0.0	0.0	0.0	0.0	0.0	0.0	0.0
Q53	0.0	0.0	0.0	0.0	0.0	0.0	0.0	0.0	0.0	0.0	0.0	0.0	0.0	5.2	3.6	0.0	0.0	0.0	0.0
C54	0.0	0.0	0.0	0.0	0.0	0.0	0.0	0.0	0.0	0.0	0.0	0.0	0.0	2.7	2.8	0.0	0.0	0.0	0.0
Q55	0.0	0.0	0.0	0.0	0.0	0.0	0.0	0.0	0.0	0.0	0.0	0.0	1.4	38.0	14.4	3.6	0.9	0.3	3.4
D56	0.0	0.0	0.0	0.0	0.0	0.0	0.0	0.0	0.0	0.0	0.0	0.0	0.0	0.0	0.1	0.0	0.0	0.0	7.8

N57	0.0	0.0	0.0	0.0	0.0	0.0	0.0	0.0	0.0	0.0	0.0	0.0	0.0	0.0	0.2	0.0	0.0	0.0	0.0
K58	0.0	0.0	0.0	0.0	0.0	0.0	0.0	0.0	0.0	0.0	0.0	0.0	0.0	0.0	0.0	0.0	0.0	0.0	0.0
K59	0.0	0.0	0.0	0.0	0.0	0.0	0.0	0.0	0.0	0.0	0.0	0.0	0.0	0.0	0.0	0.0	0.0	0.0	0.0
C60	0.0	0.0	0.0	0.0	0.0	0.0	0.0	0.0	0.0	0.0	0.0	0.0	0.0	0.0	0.0	0.0	0.0	0.0	0.0
C61	0.0	0.0	0.0	0.0	0.0	0.0	0.0	0.0	0.0	0.0	0.0	0.0	0.0	0.0	0.0	0.0	0.0	0.0	0.0
V62	0.0	0.0	0.0	0.0	0.0	0.0	0.0	0.0	0.0	0.0	0.0	0.0	0.0	0.0	0.0	0.0	0.0	0.0	0.0
F63	0.0	0.0	0.0	0.0	0.0	0.0	9.4	28.5	0.0	0.0	0.1	0.0	0.0	0.0	25.1	0.0	0.0	0.0	0.0
S64	0.0	0.0	0.0	0.0	0.0	0.0	0.0	0.0	0.0	0.0	0.0	0.0	0.0	0.0	0.0	0.0	0.0	0.0	0.0
C65	0.0	0.0	0.0	0.0	0.0	0.0	0.0	0.0	0.0	0.0	0.0	0.0	0.0	0.0	0.0	0.0	0.0	0.0	0.0
G66	0.0	0.0	0.0	0.0	0.0	0.0	0.0	0.0	0.0	0.0	0.0	0.0	0.0	0.0	0.0	0.0	0.0	0.0	0.0
K67	0.0	0.0	0.0	0.0	0.0	0.0	0.0	0.0	0.0	0.0	0.0	0.0	0.0	0.0	0.0	0.0	0.0	0.0	0.0
K68	0.0	0.0	0.0	0.0	0.0	0.1	2.9	4.4	1.0	0.0	87.0	80.6	54.7	0.1	61.6	0.0	0.0	0.0	3.0
C69	0.0	0.0	0.0	0.0	0.0	0.0	0.0	0.0	0.0	0.0	0.1	0.0	4.0	24.3	54.8	0.0	0.0	0.0	0.0
L70	0.0	0.0	0.0	0.0	0.0	0.0	0.0	0.0	0.0	0.0	0.0	0.0	0.1	0.2	13.2	0.0	0.0	0.0	0.0
D71	0.0	0.0	0.9	0.0	0.0	0.2	93.2	0.0	0.0	0.0	0.0	0.1	0.1	0.1	47.9	0.0	35.4	0.3	0.3
L72	0.0	0.0	0.9	0.0	0.0	0.0	0.0	0.0	0.0	0.0	0.1	2.4	4.8	13.2	86.0	7.4	41.5	0.4	6.3
K73	2.0	0.3	1.0	0.0	0.0	0.0	0.0	0.0	0.0	0.0	0.0	0.0	0.0	1.2	5.4	29.2	26.5	20.8	23.7
Q74	2.9	3.6	13.4	0.1	0.0	2.1	4.9	0.0	0.0	0.0	0.0	0.0	0.0	0.0	2.5	0.7	47.1	19.9	17.6
D75	0.0	0.0	0.0	0.0	0.0	0.0	0.0	0.0	0.0	0.0	0.0	0.0	0.0	0.0	0.0	0.0	0.7	0.0	2.2
V76	0.0	0.0	0.0	0.0	0.0	0.0	0.0	0.0	0.0	0.0	0.0	0.0	0.0	0.0	0.0	0.0	0.0	0.0	0.0
C77	0.0	0.0	0.0	0.0	0.0	0.0	0.0	0.0	0.0	0.0	0.0	0.0	0.0	0.0	0.0	0.0	0.0	0.0	0.0
E78	0.0	0.0	0.0	0.0	0.0	0.0	0.0	0.0	0.0	0.0	0.0	0.0	0.0	0.0	0.0	0.0	3.9	0.0	2.9
M79	0.6	1.2	19.4	1.8	0.0	0.0	45.0	0.0	1.0	0.0	0.0	0.0	0.0	0.0	0.2	0.0	56.1	0.2	2.6
P80	1.5	10.8	26.5	8.0	0.0	0.0	0.0	0.0	0.0	0.0	0.0	0.0	0.0	0.0	0.0	0.0	8.5	0.1	5.2
K81	1.8	2.8	16.9	4.2	0.0	0.0	0.3	0.0	0.0	0.0	0.0	0.0	0.0	0.0	0.0	0.0	2.5	0.0	0.5
E82	3.7	24.4	9.3	40.9	1.3	0.0	0.0	1.7	0.0	0.0	0.0	0.0	0.0	0.0	0.0	0.0	0.0	0.0	0.1
T83	20.9	15.2	13.7	1.8	0.0	0.0	0.0	0.1	0.0	0.0	0.0	0.0	0.0	0.0	0.0	0.0	0.0	0.1	2.9
G84	0.3	0.1	0.0	0.0	0.0	0.0	0.0	0.0	0.0	0.0	0.0	0.0	0.0	0.0	0.0	0.0	0.0	0.0	0.0
P85	0.0	0.0	0.0	0.0	0.0	0.0	0.0	0.0	0.0	0.0	0.0	0.0	0.0	0.0	0.0	0.0	0.0	0.0	0.0
C86	0.0	0.0	0.0	0.0	0.0	0.0	0.0	0.0	0.0	0.0	0.0	0.0	0.0	0.0	0.0	0.0	0.0	0.0	0.0
L87	0.0	0.0	0.0	0.0	0.0	0.0	0.0	0.0	0.0	0.0	0.0	0.0	0.0	0.0	0.0	0.0	0.0	0.0	0.0
A88	0.0	0.0	0.0	0.0	0.0	0.0	0.0	0.0	0.0	0.0	0.0	0.0	0.0	0.0	0.0	0.0	0.0	0.0	0.0
Y89	0.0	0.0	0.0	0.0	0.0	0.0	0.0	0.0	0.0	0.0	0.0	0.0	0.0	0.0	0.0	0.0	0.0	0.0	0.0
F90	0.0	0.0	0.0	0.0	0.0	0.0	0.0	0.0	0.0	0.0	0.0	0.0	0.0	0.0	0.0	0.0	0.0	0.0	0.0
L91	0.0	0.0	0.0	0.0	0.0	0.0	0.0	0.0	0.0	0.0	0.0	0.0	0.0	0.0	0.0	0.0	0.0	0.0	0.0
H92	0.0	0.0	0.0	0.0	0.0	0.0	0.0	0.0	0.0	0.0	0.0	0.0	0.0	0.0	0.0	0.0	0.0	0.0	0.0
W93	0.0	0.0	0.0	0.0	0.0	0.0	0.0	0.0	0.0	0.0	0.0	0.0	0.0	0.0	0.0	0.0	0.0	0.0	0.0
W94	0.0	0.0	0.0	0.0	0.0	0.0	0.0	0.0	0.0	0.0	0.0	0.0	0.0	0.0	0.0	0.0	0.0	0.0	0.0
Y95	0.0	0.0	0.0	0.0	0.0	0.0	0.0	0.0	0.0	0.0	0.0	0.0	0.0	0.0	0.0	0.0	0.0	0.0	0.0

D96	0.0	0.0	0.0	0.0	0.0	0.0	0.0	0.0	0.0	0.0	0.0	0.0	0.0	0.0	0.0	0.0	0.0	0.0	0.0
K97	0.0	0.0	0.0	0.0	0.0	0.0	0.0	0.0	0.0	0.0	0.0	0.0	0.0	0.0	0.0	0.0	0.0	0.0	0.1
K98	0.0	0.0	0.0	0.0	0.0	0.0	0.0	0.0	0.0	0.0	0.0	0.0	0.0	0.0	0.0	0.0	0.0	0.0	0.0
D99	0.0	0.0	0.0	0.0	0.0	0.0	0.0	0.0	0.0	0.0	0.0	0.0	0.0	0.0	0.0	0.0	0.0	0.0	0.0
N100	0.0	0.0	0.0	0.0	0.0	0.0	0.0	0.0	0.0	0.0	0.0	0.0	0.0	0.0	0.0	0.0	0.0	0.0	0.0
T101	0.0	0.0	0.0	0.0	0.0	0.0	0.0	0.0	0.0	0.0	0.0	0.0	0.0	0.0	0.0	0.0	0.0	0.0	0.0
C102	0.0	0.0	0.0	0.0	0.0	0.0	0.0	0.0	0.0	0.0	0.0	0.0	0.0	0.0	0.0	0.0	0.0	0.0	0.0
S103	0.0	0.0	0.0	0.0	0.0	0.0	0.0	0.0	0.0	0.0	0.0	0.0	0.0	0.0	0.0	0.0	0.0	0.0	0.0
M104	0.0	0.0	0.0	0.0	0.0	0.0	0.0	0.0	0.0	0.0	0.0	0.0	0.0	0.0	0.0	0.0	0.0	0.0	0.0
P105	0.0	0.0	0.0	0.0	0.0	0.0	0.0	0.0	0.0	0.0	0.0	0.0	0.0	0.0	0.0	0.0	0.0	0.0	0.0
V106	0.0	0.0	0.0	0.0	0.0	0.0	0.0	0.0	0.0	0.0	0.0	0.0	0.0	0.0	0.0	0.0	0.0	0.0	0.0
Y107	0.0	0.0	0.3	2.5	0.0	0.0	0.0	0.0	0.0	0.0	0.0	0.0	0.0	0.0	0.0	0.0	0.0	0.0	0.0
G108	0.0	0.0	0.0	0.0	0.0	0.0	0.0	0.0	0.0	0.0	0.0	0.0	0.0	0.0	0.0	0.0	0.0	0.0	0.0
G109	0.0	0.0	0.0	0.0	0.0	0.0	0.0	0.0	0.0	0.0	0.0	0.0	0.0	0.0	0.0	0.0	0.0	0.0	0.0
C110	0.1	1.0	0.0	0.0	0.0	0.0	0.0	0.3	0.0	0.0	0.0	0.0	0.0	0.0	0.0	0.0	0.0	0.0	0.0
Q111	17.7	29.4	16.0	51.5	24.2	0.0	0.0	12.6	12.6	0.1	0.1	0.0	0.0	0.0	0.0	0.0	0.1	0.0	0.3
G112	0.0	0.4	11.8	42.8	0.0	0.0	7.9	36.1	0.2	0.0	0.0	0.0	0.0	0.0	0.0	0.0	0.0	0.0	0.0
N113	1.2	2.1	42.9	40.6	0.0	0.0	66.2	5.4	0.0	0.0	0.0	0.0	0.0	0.0	0.0	0.0	11.9	0.0	1.3
N114	0.0	0.0	3.7	2.6	0.0	0.0	94.5	57.3	0.0	0.0	0.4	0.0	0.0	0.0	15.5	0.0	0.2	0.0	0.0
N115	0.0	0.0	0.3	0.0	0.0	0.0	88.5	0.0	0.0	0.0	0.0	0.0	0.0	0.0	0.9	0.0	7.1	0.0	0.3
N116	0.0	0.0	0.0	0.0	0.0	0.0	0.0	0.0	0.0	0.0	0.0	0.0	0.0	0.0	0.0	0.0	0.0	0.0	0.0
F117	0.0	0.0	0.0	0.0	0.0	0.0	0.0	0.0	0.0	0.0	0.0	0.0	0.0	0.0	0.0	0.0	0.0	0.0	0.0
Q118	0.0	0.0	0.0	0.0	0.0	0.0	0.0	0.0	0.0	0.0	0.0	0.0	0.0	0.0	0.0	0.0	0.0	0.0	0.0
S119	0.0	0.0	0.0	0.0	0.0	0.0	0.0	0.0	0.0	0.0	0.0	0.0	0.0	0.0	0.0	0.0	0.0	0.0	0.0
K120	0.0	0.0	0.0	0.0	0.0	0.0	0.0	0.0	0.0	0.0	0.0	0.0	0.0	0.0	0.0	0.0	0.0	0.0	0.0
A121	0.0	0.0	0.0	0.0	0.0	0.0	0.0	0.0	0.0	0.0	0.0	0.0	0.0	0.0	0.0	0.0	0.0	0.0	0.0
N122	0.0	0.0	0.0	0.0	0.0	0.0	0.0	0.0	0.0	0.0	0.0	0.0	0.0	0.0	0.0	0.0	0.0	0.0	0.0
C123	0.0	0.0	0.0	0.0	0.0	0.0	0.0	0.0	0.0	0.0	0.0	0.0	0.0	0.0	0.0	0.0	0.0	0.0	0.0
L124	0.0	0.0	0.0	0.0	0.0	0.0	0.0	0.0	0.0	0.0	0.0	0.0	0.0	0.0	0.0	0.0	0.0	0.0	0.0
N125	0.0	0.0	0.0	0.0	0.0	0.0	0.0	0.0	0.0	0.0	0.0	0.0	0.0	0.0	0.0	0.0	0.0	0.0	0.0
T126	0.0	0.0	0.0	0.0	0.0	0.0	0.0	0.0	0.0	0.0	0.0	0.0	0.0	0.0	0.0	0.0	0.0	0.0	0.0
C127	0.0	0.0	0.0	0.0	0.0	0.0	0.0	0.0	0.0	0.0	0.0	0.0	0.0	0.0	0.0	0.0	0.0	0.0	0.0
K128	0.0	0.0	0.0	0.0	0.0	0.0	0.0	0.0	0.0	0.0	0.0	0.0	0.0	0.0	0.0	0.0	0.0	0.0	0.0
N129	0.0	0.0	0.0	0.0	0.0	0.0	0.0	0.0	0.0	0.0	0.0	0.0	0.0	0.0	0.0	0.0	0.0	0.0	0.0
K130	0.0	0.0	0.0	0.0	0.0	0.0	0.0	0.0	0.0	0.0	0.0	0.0	0.0	0.0	0.0	0.0	0.0	0.0	0.0
R131	0.0	0.0	0.0	0.0	0.0	0.0	0.0	0.0	0.0	0.0	0.0	0.0	0.0	0.0	0.0	0.0	0.0	0.0	1.2
F132	0.0	0.0	0.0	0.0	0.0	0.0	0.0	0.0	0.0	0.0	0.0	0.0	0.0	0.0	0.0	0.0	0.0	0.0	0.0
P133	0.0	0.0	0.0	0.0	0.0	0.0	0.0	0.0	0.0	0.0	0.0	0.0	0.0	0.0	0.0	0.0	0.0	0.0	0.0

Supplementary Table S4. Intermolecular H-bonds (≤3.5 Ä) in EPPIN/EP055 complexes during molecular dynamic simulations.

% Interaction overtime				
EPPIN residue	EP055/EPPIN complex (500 ns)			EP012/EPPIN complex (100 ns)
	Cla Docking 1	Cla Docking 2	Clc Docking 1	
P22	0.0	0.0	0.0	0.0
G23	0.0	0.0	0.0	0.0
L24	0.0	0.0	0.0	0.0
T25	0.0	0.0	0.0	0.0
D26	0.0	0.0	0.0	0.0
W27	0.0	0.0	0.0	0.0
K28	0.0	0.0	0.0	0.0
F29	0.0	0.0	0.0	0.0
P30	0.0	0.0	0.0	0.0
R31	0.0	0.0	0.0	0.0
R32	0.0	0.0	0.0	0.0
C33	0.0	0.0	0.0	0.0
P34	0.3	0.2	0.7	0.09
K35	0.3	0.0	16.6	0.05
I36	2.1	0.5	30.2	10.69
R37	97.4	1.4	50.4	89.68
E38	5.2	0.0	1.7	0.02
E39	62.0	0.7	0.2	28.88
C40	0.3	0.2	0.4	0.0
E41	1.1	71.5	8.9	0.12
F42	4.4	88.8	30.3	0.00
Q43	5.0	91.8	27.3	0.0
E44	0.0	0.1	2.7	0.0
R45	0.6	52.3	13.1	0.23
D46	0.0	0.0	0.0	0.0
V47	8.1	25.6	7.4	1.16
C48	0.0	0.0	0.0	0.0
T49	0.0	0.0	0.0	0.0
K50	0.0	0.0	0.0	0.0
D51	0.0	0.0	0.0	0.0
R52	0.0	0.0	0.0	0.0
Q53	0.0	0.0	0.0	0.0
C54	0.0	0.9	1.3	0.0
Q55	0.7	22.1	2.9	0.18
D56	0.0	2.7	4.1	0.0
N57	0.6	0.0	43.6	1.54
K58	0.0	0.0	0.0	0.0
K59	0.0	0.0	0.0	0.0
C60	0.0	0.0	0.0	0.0
C61	0.0	0.0	0.0	0.0
V62	0.0	0.0	0.0	0.0
F63	94.2	97.4	3.7	63.97
S64	0.0	0.0	0.0	0.01
C65	0.0	0.0	0.0	0.0
G66	0.0	0.0	0.0	0.0
K67	0.0	0.0	0.0	0.0
K68	60.1	99.7	20.1	44.79
C69	68.9	100.0	45.3	50.29
L70	1.2	58.6	1.4	1.27
D71	100.0	100.0	10.2	99.95
L72	7.7	12.8	0.0	28.70

K73	0.0	67.4	0.0	19.34
Q74	42.4	96.5	43.5	70.58
D75	2.9	0.0	83.8	10.52
V76	1.9	2.7	1.2	1.78
C77	0.0	0.0	0.0	0.0
E78	0.0	0.0	100.0	0.0
M79	12.9	0.1	61.3	28.75
P80	0.5	0.0	43.2	0.03
K81	0.0	0.0	7.7	0.27
E82	0.5	0.0	2.8	0.53
T83	1.8	0.0	3.6	0.0
G84	0.1	0.0	5.1	0.0
P85	0.1	0.0	0.0	0.0
C86	0.0	0.0	0.0	0.0
L87	0.0	0.0	0.0	0.0
A88	0.0	0.0	0.0	0.0
Y89	0.0	0.0	0.0	0.0
F90	0.0	0.0	0.0	0.0
L91	0.0	0.0	0.0	0.0
H92	0.0	0.0	0.0	0.0
W93	0.0	0.0	0.0	0.0
W94	0.0	0.0	0.7	0.0
Y95	0.0	0.0	0.0	0.0
D96	0.0	0.0	0.0	0.0
K97	0.0	0.0	46.5	0.0
K98	0.0	0.0	0.0	0.0
D99	0.0	0.0	0.0	0.0
N100	0.0	0.0	0.0	0.0
T101	0.0	0.0	0.0	0.0
C102	0.0	0.0	0.0	0.0
S103	0.0	0.0	0.0	0.0
M104	0.0	0.0	0.0	0.0
P105	0.0	0.0	0.0	0.0
V106	0.0	0.0	0.0	0.0
Y107	0.0	0.0	0.0	0.0
G108	0.0	0.0	0.0	0.0
G109	0.0	0.0	0.0	0.0
C110	0.0	0.0	0.0	0.0
Q111	0.4	0.0	4.4	1.42
G112	74.2	0.0	1.7	58.58
N113	97.3	40.3	19.4	90.72
N114	98.6	30.7	0	92.56
N115	38.3	3.1	3.2	76.43
N116	0.0	0.0	0.0	0.0
F117	0.0	0.0	0.0	0.0
Q118	0.0	0.0	0.0	0.0
S119	0.0	0.0	0.0	0.0
K120	0.0	0.0	0.0	0.0
A121	0.0	0.0	0.0	0.0
N122	0.0	0.0	0.0	0.0
C123	0.0	0.0	0.0	0.0
L124	0.0	0.0	0.0	0.0
N125	0.0	0.0	0.0	0.0
T126	0.0	0.0	0.0	0.0
C127	0.0	0.0	0.0	0.0
K128	0.0	0.0	0.3	0.0
N129	0.0	0.0	0.0	0.0

K130	0.0	0.0	50.0	0.0
R131	0.0	0.0	6.0	0.0
F132	0.0	0.0	0.0	0.0
P133	0.0	0.0	0.0	0.0

Supplementary Table S5. Interaction time in EPPIN/SEMG1-E2Q complexes during Orientations of Proteins in Membranes.

% Interaction time	
EPPIN residue	EPPIN/SEMG1-E2Q complex
P22	81.3
G23	99.9
L24	100.0
T25	99.9
D26	98.9
W27	57.7
L28	86.6
F29	99.6
P30	26.6
R31	23.8
R32	7.8
C33	0.0
P34	0.0
K35	7.5
I36	0.0
R37	0.3
E38	0.0
E39	3.5
C40	9.8
E41	0.0
F42	0.0
Q43	0.0
E44	0.0
R45	0.0
D46	0.0
V47	0.8
C48	0.2
T49	5.2
K50	35.2
D51	2.1
R52	92.0
Q53	34.6
C54	10.5
Q55	42.4
D56	33.0
N57	0.0
K58	37.7
K59	0.3
C60	0.0
C61	0.0
V62	0.0
F63	0.0
S64	0.0
C65	0.0
G66	0.0
K67	0.2
K68	0.0
C69	0.0
L70	0.0
D71	0.0
L72	5.7
K73	49.2
Q74	45.7
D75	40.5
V76	0.0
C77	0.0
E78	56.4
M79	7.2
P80	14.3
K81	0.0
E82	0.0
T83	0.0
G84	0.0
P85	0.0
C86	0.0
L87	0.0
A88	0.0
Y89	0.0
F90	0.0
L91	0.0
H92	0.0
W93	0.0
W94	2.7
Y95	98.3
D96	66.9
K97	96.8
K98	99.1
D99	96.6
N100	99.9
T101	12.4
C102	0.4
S103	0.0
M104	0.0
P105	0.0
V106	0.0
Y107	0.0
G108	0.0
G109	0.0
C110	0.0
Q111	0.0
G112	0.0
N113	0.0
N114	0.0
N115	0.0
N116	0.0
F117	0.0
Q118	0.0
S119	0.0
K120	1.3
A121	1.0
N122	0.0
C123	0.0
L124	88.8
N125	71.2
T126	4.7
C127	40.7
K128	99.1
N129	78.3
K130	99.9
R131	99.7
F132	100.0
P133	100.0
